# The APC/C targets the Cep152–Cep63 complex at the centrosome to regulate mitotic spindle assembly

**DOI:** 10.1242/jcs.259273

**Published:** 2022-01-18

**Authors:** Thomas Tischer, Jing Yang, David Barford

**Affiliations:** MRC Laboratory of Molecular Biology, Francis Crick Avenue, Cambridge CB2 0QH, UK

**Keywords:** APC/C, Centrosome, Cep152, Ubiquitin, Microtubules, Mitosis

## Abstract

The control of protein abundance is a fundamental regulatory mechanism during mitosis. The anaphase-promoting complex/cyclosome (APC/C) is the main protein ubiquitin ligase responsible for the temporal regulation of mitotic progression. It has been proposed that the APC/C might fulfil other functions, including assembly of the mitotic spindle. Here, we show that the APC/C localizes to centrosomes, the organizers of the eukaryotic microtubule cytoskeleton, specifically during mitosis. Recruitment of the APC/C to spindle poles requires the centrosomal protein Cep152, and we identified Cep152 as both an APC/C interaction partner and an APC/C substrate. Previous studies have shown that Cep152 forms a complex with Cep57 and Cep63. The APC/C-mediated ubiquitylation of Cep152 at the centrosome releases Cep57 from this inhibitory complex and enables its interaction with pericentrin, a critical step in promoting microtubule nucleation. Thus, our study extends the function of the APC/C from being a regulator of mitosis to also acting as a positive governor of spindle assembly. The APC/C thereby integrates control of these two important processes in a temporal manner.

## Abstract (precis)

**Summary:** The ubiquitin ligase APC/C is required for anaphase execution and localizes to the centrosome during mitosis. Its function is to remove Cep152, an inhibitor of microtubule nucleation, from the centrosome.

## INTRODUCTION

During mitosis, a mother cell divides into two daughter cells that both inherit identical genetic material. This process needs to be tightly controlled to ensure that chromosomes are equally distributed. Mitosis is therefore regulated by multiple mechanisms that work together to coordinate a successful cell division. One important regulatory mechanism is ubiquitin-mediated protein degradation, which controls the abundance of different cell cycle regulators during mitosis. The anaphase-promoting complex/cyclosome (APC/C) is a multi-subunit E3 ubiquitin ligase governing this process. APC/C activity is coordinated by the correct attachment of chromosomes to the mitotic spindle. During recent years, the molecular structure and mechanisms of the APC/C have been defined (Alfieri et al., 2017; Watson et al., 2019); however, relatively little is known about the role of its intracellular localization. The APC/C has been observed at kinetochores during an active spindle assembly checkpoint (SAC) (Acquaviva et al., 2004) and at chromosomes (Sivakumar et al., 2014). Additionally, studies suggest that localized protein degradation plays an important role during mitosis. The APC/C substrate cyclin B, for example, has been reported to be degraded first at the spindle poles (Clute and Pines, 1999; Huang and Raff, 1999). Consequently, the APC/C has been shown to localize to spindle poles, with this recruitment being dependent on the protein NuMA (also known as NUMA1) and the motor protein dynein (Ban et al., 2007; Tugendreich et al., 1995). Recently, it has also been demonstrated in *Drosophila* that the APC/C co-activator Cdh1 (also known as Fzr in *Drosophila*) is recruited to centrosomes by the centrosomal protein Cep192 (also known as Spd2 in *Drosophila*; Meghini et al., 2016). It has therefore been speculated that the APC/C could be directly involved in spindle assembly.

Centrosomes are cellular organelles and the major organizers of the microtubule cytoskeleton in eukaryotic cells (Gönczy, 2012). In higher eukaryotes, centrosomes consist of two cylindrical shaped structures named centrioles, which are embedded in a protein-rich matrix, the pericentriolar material (PCM). Centrosomes are duplicated in S phase parallel to but independently from the chromatids, and this process is regulated by Polo-like kinase 4 (Plk4) together with additional proteins such as Cep192, Cep152 and Cep63 (Brown et al., 2013; Nigg and Holland, 2018; Stearns, 2001). Centrosomes dynamically recruit additional proteins around them to form the PCM, including pericentrin (PCNT), Cdk5rap2 (also known as Cep215) and γ-tubulin (γTub), that are required for microtubule anchorage and nucleation (Gupta and Pelletier, 2017; Kim et al., 2019; Kim and Rhee, 2014; Thawani et al., 2018; Wieczorek et al., 2020), and consequently for mitotic spindle formation. The PCM increases in size before mitosis (Lawo et al., 2012; Sonnen et al., 2012; Watanabe et al., 2019; Woodruff et al., 2014), and proteins that are required for centriole duplication have also been implicated in pathways that lead to microtubule nucleation. These include Cep192, as well as Cep63 and Cep57, which recruit Cep152 to the centrosome (Meitinger et al., 2020; Watanabe et al., 2020; Wei et al., 2020). Cep57 has additionally been implicated in recruiting PCNT (Watanabe et al., 2019).

In this study, we investigate the localization and function of the APC/C at spindle poles and its role in mitotic spindle assembly. Using a combination of super-resolution and confocal microscopy, cell biology and biochemical assays, we show that during mitosis the APC/C is localized to the PCM and is organized into a ring-like structure. We establish that the main interacting protein responsible for the localization of the APC/C to the centrosome is Cep152, which we additionally show to be a novel APC/C substrate. We propose that Cep152 is locally ubiquitylated by the APC/C at the centrosome during mitosis, which in turn decreases the localization of the APC/C itself at the centrosome. This negative feedback loop controlling APC/C localization is required for proper assembly of the mitotic spindle. Stabilization of a Cep152 mutant that cannot be targeted by the APC/C results in reduced microtubule nucleation and increased chromosome mis-segregation. We reveal that the mechanism behind this phenotype is a sequestering of the PCNT-binding protein Cep57 into an inhibitory complex consisting of Cep152–Cep63–Cep57 (Lukinavičius et al., 2013). Once Cep152 auto-regulates its reduction at the centrosome by APC/C-mediated ubiquitylation, levels of Cep63 are also reduced at the centrosome and Cep57 is liberated. This leads to increased recruitment of PCNT, aiding microtubule nucleation. Hence, our work shows for the first time a role of Cep152 during mitosis and extends the function of the APC/C from being a critical director of cell cycle progression, to additionally also functioning as a positive regulator of mitotic spindle assembly.

## RESULTS

### The APC/C is a component of the mitotic centrosome

**Fig. 1.**
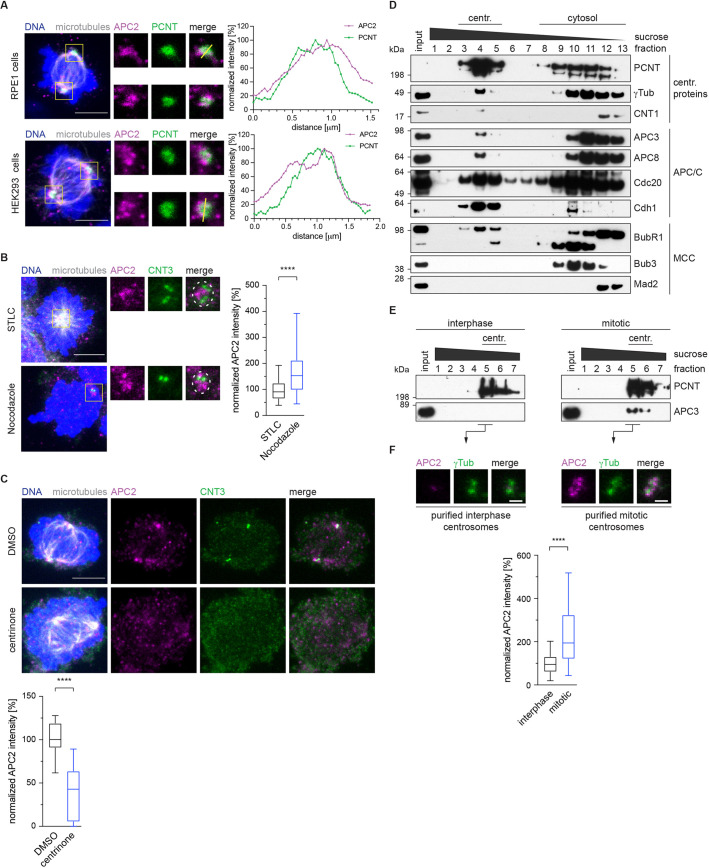
**The APC/C is a component of the mitotic centrosome.** (A) Mitotic RPE1 or HEK293 cells were pre-extracted, fixed in formaldehyde (top) or ice-cold methanol (bottom) and stained with the indicated antibodies. Boxes indicate regions shown in enlarged images. A line scan with a 10-pixel-wide line was performed at the indicated positions, and the normalized values were plotted (right). Data shown are representative of three experiments. (B) HEK293 cells were treated with STLC or nocodazole to induce a prometaphase arrest. Cells were pre-extracted, fixed in formaldehyde and stained with the indicated antibodies (CNT3, centrin-3). Boxes indicate regions shown in enlarged images. The intensity of APC2 at the centrosome was measured inside the dotted circle and normalized against the signal intensity in the presence of STLC (right). *N* cells=91 (STLC) and 84 (nocodazole). (C) HEK293 cells were treated with centrinone to deplete their centrosomes. Cells were pre-extracted, fixed in formaldehyde and stained with the indicated antibodies. The intensity of APC2 throughout the whole cell was measured and normalized against the signal intensity in DMSO (bottom). *N* cells=11 (DMSO control) and 14 (centrinone). In A–C, microtubules were labelled using an α-tubulin antibody, and DNA was stained with DAPI. (D) Centrosomes were purified from mitotic HEK293 cells using a sucrose gradient (centr., centrosomal). The eluted fractions were immunoblotted against the indicated proteins. The top shows PCNT, γTub, and centrin-1 (CNT1) as centrosomal proteins, the middle shows several APC/C components, and MCC proteins are shown at the bottom. Blots are representative of four experiments. (E) Centrosomes were purified in parallel from interphase HEK293 cells and mitotic HEK293 cells using a sucrose gradient. The first elution fractions were blotted to detect PCNT as a centrosomal marker and APC3. Blots are representative of three experiments. (F) Centrosomes from E were fixed on a coverglass and stained with the indicated antibodies (top). The intensity of APC2 was measured as indicated in B and normalized against the signal intensity on interphase centrosomes (bottom). *N* centrosomes=142 (interphase) and 123 (mitotic). Box plots show the median (line), 25–75% range (box) and 5–95% range (whiskers). ****P<0.0001 (Mann–Whitney *U*-test). Scale bars: 5 µm in A–C, 1 μm in F.

It has previously been shown that the APC/C is localized around spindle poles in mitosis (Ban et al., 2007; Tugendreich et al., 1995). Using commercial antibodies and a similar fixation and staining protocol, we were able to confirm these data by testing three different APC/C subunits, namely APC2 (also known as ANAPC2), APC3 (CDC27) and APC8 (CDC23) (Fig. 1A; Fig. S1A). APC/C core-subunits are rarely present in isolation, which means the independent detection of three subunits of the complex established the presence of the whole E3 ubiquitin ligase (Passmore, 2004; Vodermaier et al., 2003). The APC/C showed a clear colocalization with PCNT, a protein enriched in the PCM of centrosomes, and this was consistent between different cell lines and with different APC/C subunits (Fig. 1A; Fig. S1A). Additionally, the APC/C also appeared to localize to microtubules around the spindle pole; however, depolymerization of microtubules using nocodazole did not abolish APC/C localization close to centrosomes (Fig. 1B). We decided to only continue with the APC2 antibody for immunofluorescence, because it gave the most consistent staining. The specificity of the APC2 antibody was confirmed by depletion of the APC2 subunit using siRNA, after which the signal disappeared in immunoblotting and immunofluorescence experiments (Fig. S1B–D). To characterize the spindle pole localization of the APC/C in more detail, we decided to test whether centrosomes are required for this localization. Treatment of cells with the Plk4 inhibitor centrinone depletes cells of their centrosomes (Wong et al., 2015), but human embryonic kidney (HEK) cells continue to divide even without the organelle present (Gheiratmand et al., 2019). In the absence of centrosomes, APC/C localization at spindle poles was greatly diminished (Fig. 1C), arguing that the centrosome itself is needed to localize the APC/C to spindle poles in mitosis. Based on these findings, centrosomes were purified from mitotic cells (Fig. 1D) using a sucrose density gradient. Heavy complexes such as centrosomes elute early from the gradient. This purification showed a clear co-elution of APC/C subunits with centrosomal fractions (Fig. 1D; co-elution of the APC2 subunit with centrosomal fractions can be seen in Fig. S3C). Additionally, we also detected the APC/C co-activators Cdc20 and Cdh1 (also known as FZR1), which have been found previously to localize to the centrosomes (Gambarotto et al., 2019; Kallio et al., 2002; Meghini et al., 2016; Zhou et al., 2003). We identified BubR1 (also known as BUB1B) in centrosomal fractions but did not observe co-elution of the other SAC proteins Mad2 (MAD2L1) or Bub3 (Fig. 1D). This is in agreement with a previous observation that BubR1 localizes to centrosomes independently of the mitotic checkpoint complex (MCC; Izumi et al., 2009). We also purified centrosomes from mitotic cells (arrested using the microtubule poison nocodazole) and cycling interphase cells to compare the levels of APC/C present during different cell cycle stages. This experiment revealed a significant increase in the APC/C signal in mitotic cells, indicating that the APC/C localized to centrosomes specifically in mitosis (Fig. 1E,F). In summary, our data clearly show the colocalization of APC/C subunits with the mitotic centrosome, using multiple criteria of immunofluorescence and the co-elution of different APC/C subunits with the purified centrosome. The localization requires intact centrosomes but not microtubules. This argues that the whole E3 ubiquitin ligase complex is present and, in strong agreement with previous data (Ban et al., 2007; Meghini et al., 2016; Tugendreich et al., 1995), we therefore confirm the APC/C as a component of the mitotic centrosome.

### The APC/C localizes inside the PCM towards the proximal end of the centriole

We established that the APC/C is present at mitotic centrosomes. To better understand the localization of the APC/C at this organelle, we performed direct stochastic optical reconstruction microscopy (dSTORM) (Betzig et al., 2006; Rust et al., 2006) of early prometaphase-arrested cells. In two dimensions, the APC/C localized in a regular, spot-like, circular pattern with a diameter of 524±77 nm (mean±s.d.; Fig. S2A,B). The interphase PCM consists of different layers or rings (Sonnen et al., 2012; Watanabe et al., 2019; Woodruff et al., 2014), and based on previous measurements, this placed the APC/C towards the outer region of the intermediate PCM, close to proteins such as Cep152, Cdk5rap2, and γTub (Lawo et al., 2012; Sonnen et al., 2012). This is in agreement with our results showing colocalization of the APC/C with the PCM protein PCNT (Fig. 1A,B). Analysis of single centrosomal APC/C dots showed a diameter of 76±28 nm (Fig. S2D), which corresponds closely to the calculated size of a single APC/C molecule of 35 nm plus two times 15 nm for the primary and secondary antibodies. To validate this observation, we used human APC/C purified from insect cells and performed 2D-dSTORM imaging using the same conditions. The measured diameter in this case was 69±20 nm (Fig. S2C,D), indicating that the APC/C dots at the centrosome very likely represent only one or a maximum of two APC/Cs. Since there are only two centrosomes in the cell, this observation suggests that only a very small proportion of APC/C is present at the centrosome. To understand the spatial organization of the APC/C inside the PCM, two-colour 3D-dSTORM was performed. Marker proteins with a known centrosomal localization were used as reference points. The APC/C was observed to accumulate mostly in one plane around the centriolar lumen marker centrin-3 (Fong et al., 2014; Paoletti et al., 1996) (Fig. S2E, Movies 1 and 2). This indicated that the APC/C strongly localized either at the proximal or distal end of the centrosome. Using C-NAP1 (also known as the Cep250) as a marker for the proximal end of centrosomes showed that the APC/C and C-NAP1 reside within a similar *z*-plane (Fig. S2F, Movies 3 and 4), which leads to the conclusion that the APC/C is localized around the proximal end. In summary, we show that individual APC/Cs accumulate in a symmetrical ring-like pattern surrounding the proximal end of the centrosome within the intermediate PCM (Fig. S2G).

### The APC/C interacts with several other centrosomal proteins and disappears from the centrosome during mitosis

**Fig. 2.**
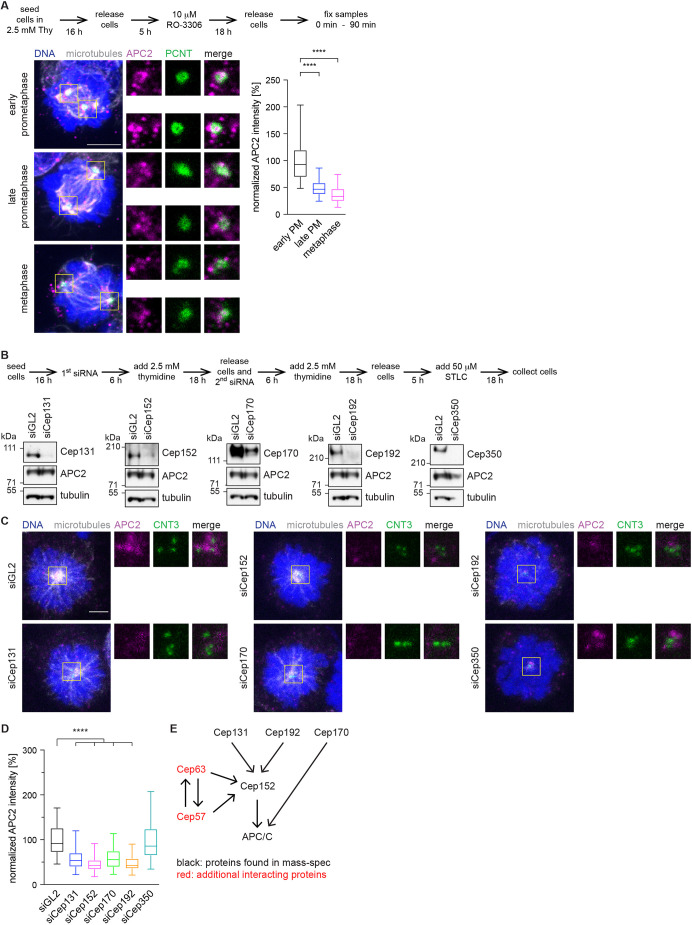
**Depletion of centrosomal proteins leads to loss of the APC/C from the centrosome.** (A) HEK293 cells were treated according to the timeline (top; Thy, thymidine) and stained to detect the indicated proteins. Microtubules were labelled using an α-tubulin antibody, and DNA was stained with DAPI. Boxes indicate regions shown in enlarged images. The fluorescence intensity of APC2 at the centrosome was measured as indicated in Fig. 1B and normalized against the intensity in early prometaphase (PM). *N* cells=77 (early PM), 130 (late PM), 73 (metaphase). (B) HEK293 cells were treated according to the timeline (top). Cells were depleted of the centrosomal proteins chosen for further analysis using siRNA (siGL2, control siRNA). Whole-cell lysates from STLC arrested, mitotic cells were immunoblotted against the indicated proteins. Tubulin serves as a loading control. Blots are representative of three experiments. (C) Cells were treated as in B and stained against the indicated proteins (CNT3, centrin-3). Microtubules were labelled using an α-tubulin antibody, and DNA was stained with DAPI. Boxes indicate regions shown in enlarged images. (D) Quantification of the APC2 intensity at the centrosome from cells shown in C. The intensity was measured as indicated in Fig. 1B and normalized against the siGL2 control. *N* cells=140 (siGL2), 109 (siCep131), 52 (siCep152), 86 (siCep70), 51 (siCep192), 63 (siCep350). (E) Interaction model of the different centrosomal proteins with each other and with the APC/C. Box plots show the median (line), 25–75% range (box) and 5–95% range (whiskers).****P<0.0001 (simple one-way ANOVA with Dunnett's multiple comparison test). Scale bars: 5 µm.

After fixation and staining of HEK cells during different phases of mitosis, we observed that the APC/C is highly accumulated at the centrosome during early prometaphase, when cells possess mostly a monopolar spindle. With further progression through mitosis (late prometaphase) the APC/C signal at the centrosome decreased, until only about half of the initial signal intensity remained in metaphase (Fig. 2A). Based on these data, we wondered whether the APC/C is involved in mitotic spindle assembly and, if so, which proteins might interact with the APC/C during its localization at the centrosome. To address this question, we performed proximity labelling and mass spectrometry. For this, we tagged the APC/C subunits APC2 and APC3 independently with BioID2 under the control of a tetracycline-inducible promotor. This improved version of the biotin ligase BirA is constitutively active (Kim et al., 2016). Upon addition of tetracycline, expression of the transgene was induced, but labelling of proteins with the biotin moiety was observed only after addition of exogenous biotin (Fig. S3A). Immunoprecipitation from whole-cell lysates using streptavidin confirmed the successful incorporation of the BioID2-tagged APC/C subunits into the APC/C, as well as the proficient labelling with biotin (Fig. S3B). To specifically enrich for centrosomal proteins that interact with the APC/C, we purified centrosomes from our BioID2 cell lines (Fig. S3C). We noticed that most of the endogenous APC2 in our centrosomal fractions was replaced by the BioID-tagged protein, suggesting efficient incorporation and subsequent labelling with biotin (Fig. S3D). The fraction of BioID-tagged APC3 found at the centrosome seemed to be lower compared to that of APC2, and the subsequent streptavidin pulldown confirmed weaker biotin labelling of these samples (Fig. S3C,D). The streptavidin pulldown from the purified centrosomes was then analysed using mass spectrometry. Label-free quantification (Zybailov et al., 2006) of the different datasets identified several centrosomal proteins that showed a strong enrichment in both tagged cell lines over three independent repeats (Fig. S3E, Tables S1 and S2). These included Cep131, Cep152, Cep170, Cep192, Cep350 and PCM1. Subsequent analysis focused on the Cep proteins, but not PCM1, since centriolar satellites are dissolved during mitosis (Kubo and Tsukita, 2003). It was therefore excluded as being a false positive, probably caused by contamination with interphase cells in our sample preparation.

To investigate the relationship between the identified centrosomal proteins and the APC/C, we separately depleted the different Cep proteins (Fig. 2B) and measured the intensity of the APC/C at the centrosome in mitosis (Fig. 2C,D). During the time span of our experiments, centrioles were not depleted in the cells, which excludes the possibility that a change of the APC/C signal is due the absence of centrosomes (Figs 1C, 2C). To circumvent potential problems during mitotic spindle assembly that could arise after depletion of centrosomal proteins (Meitinger et al., 2020; Watanabe et al., 2020), we decided to arrest all cells in early prometaphase using the kinesin 5-like (Eg5, also known as KIF11) inhibitor STLC. Removal of most Cep proteins resulted in various degrees of reduced APC/C intensity, except for Cep350, where APC/C intensity at the centrosome remained unchanged (Fig. 2C,D). Subsequent analysis focused on Cep131, Cep152, Cep170 and Cep192, and excluded Cep350.

**Fig. 3.**
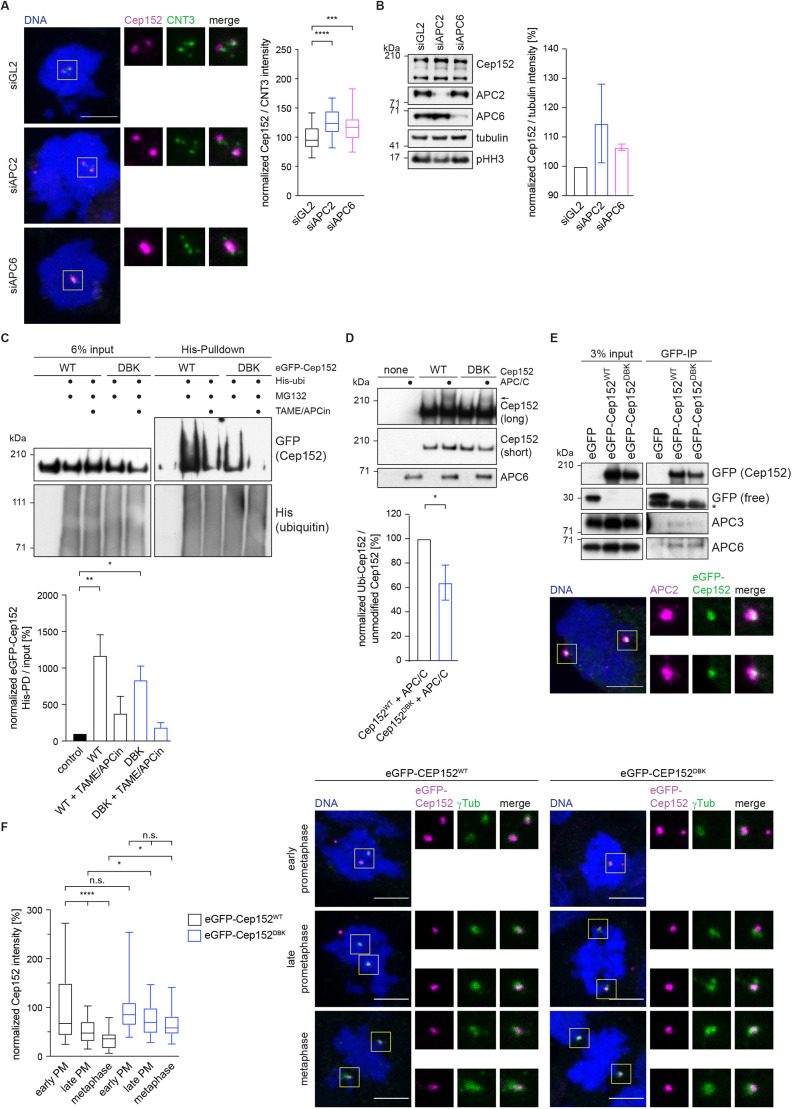
**Cep152 is a substrate of the APC/C.** (A) HEK293 cells were treated with siRNA against APC2, APC6 or GL2 (control) and arrested in mitosis with STLC according to the timeline shown in Fig. 2A. Cells were stained against the indicated proteins (CNT3, centrin-3) and DNA was labelled with DAPI. Boxes indicate regions shown in enlarged images. The fluorescence intensity of Cep152 at the centrosome was measured as indicated in Fig. 1B and normalized against the GL2 control (right). *N* cells=60 (siGL2), 60 (siAPC2), 62 (siAPC6). ***P=0.0002; ****P<0.0001 (simple one-way ANOVA with Dunnett's multiple comparison test). (B) HEK293 cells were treated as described in A, and whole-cell lysates were immunoblotted against the indicated proteins (left; pHH3, phosphorylated histone H3). The intensity of the Cep152 bands was measured and normalized to tubulin. The data from three experiments were normalized against their corresponding intensities from the siGL2 condition and plotted as mean±s.d. (right). (C) HEK293 cells expressing eGFP–Cep152^WT^ (WT) or eGFP–Cep152^DBK^ (DBK) were transfected with a plasmid expressing His–ubiquitin (His-ubi) and treated with the indicated reagents. His-tagged ubiquitin was pulled down from the cell lysate, and the eluate was blotted against the indicated proteins (top). The intensity values of the His-modified eGFP–Cep152 bands in the pulldown were normalized against the values from the corresponding input lanes (bottom) and plotted as mean±s.d. *N* immunoblots=2. *P=0.03; **P=0.007 (simple one-way ANOVA with Dunnett's multiple comparison test). (D) eGFP–Cep152 was immunoprecipitated from HEK293 cells expressing the indicated constructs. The eluate was used for *in vitro* ubiquitylation assays by incubation with purified APC/C. Arrow marks the ubiquitylated band (top). Short exposure was 30 s, long exposure was >5 min. The intensity of the ubiquitylated bands was measured, normalized to the corresponding unmodified band and plotted as mean±s.d. (bottom). *N* immunoblots=3. *P<0.05 (two-tailed paired Student's *t*-test). (E) Top: eGFP-tagged Cep152 proteins were immunoprecipitated from HEK293 cells expressing the indicated proteins. The co-eluted proteins were probed by immunoblotting. Asterisk marks unspecific bands. Blots are representative of two experiments. Bottom: Mitotic HEK293 cells expressing eGFP–Cep152^WT^ were stained against the indicated proteins, and DNA was labelled with DAPI. Boxes indicate regions shown in enlarged images. Images are representative of two experiments. (F) HEK293 FlpIn T-Rex cell lines expressing eGFP–Cep152^WT^ or eGFP–Cep152^DBK^ were treated as shown in Fig. 2A and stained against the indicated proteins (right). DNA was labelled with DAPI. Boxes indicate regions shown in enlarged images. The fluorescence intensity of eGFP–Cep152 at the centrosome was measured as indicated in Fig. 1B and normalized against the intensity of early prometaphase (PM) cells of the corresponding cell line (left). *N* cells=44 (WT, early PM), 34 (DBK, early PM), 41 (WT, late PM), 61 (DBK, late PM), 33 (WT, metaphase), 38 (DBK, metaphase). *P<0.05; ****P<0.0001; n.s., not significant (one-way ANOVA followed by Tukey's multiple comparison test). Box plots in A and F show the median (line), 25–75% range (box) and 5–95% range (whiskers). Scale bars: 5 µm.

Some of the Cep proteins are known to interact with and depend on each other for their centrosomal localization (Kim et al., 2013; Kodani et al., 2015; Sonnen et al., 2013). To generate a small-scale dependency network, we measured the intensity of each Cep protein under the depletion conditions of the other proteins (Fig. S4A–D). As expected, the fluorescence intensity of each protein was reduced when it was depleted itself, confirming the specificity of the staining. Cep131, Cep170 and Cep192 localized independently to the centrosome and did not depend on any of the other proteins; however, Cep152 intensity at the centrosome was reduced in the absence of Cep131 and Cep192 (Fig. S4B), consistent with previous data (Kim et al., 2013; Kodani et al., 2015; Sonnen et al., 2013). It therefore seems likely that the reduced APC/C intensity upon depletion of Cep131 and Cep192 is a secondary effect due to the absence of Cep152 in these conditions (Fig. 2C,D). Based on these data, indicating that Cep170 appeared to belong to a different dependency network, we excluded Cep170 from further analysis. Immunoprecipitation and immunofluorescence from cell lines expressing inducible eGFP-tagged Cep152 showed that it interacts and colocalizes with the APC/C (Fig. 3E), validating our BioID and mass spectrometry data. However, we cannot exclude that the Cep152-mediated APC/C interaction with the centrosome could also be indirect and involve additional centrosomal proteins not detected in our mass spectrometry (Fig. 2E). These include Cep57 and Cep63, which form a stable complex with Cep152 (Lukinavičius et al., 2013), as we discuss later. In conclusion, we show that Cep152 is the main protein that directly or indirectly interacts with the APC/C at the centrosome, with smaller contributions from other proteins.

### Cep152 is a substrate of the APC/C

Curiously, we observed that upon depletion of the APC/C by siRNA treatment, Cep152 protein levels detected by immunofluorescence at the centrosome increased (Fig. 3A), while at the same time the intensity of spindle microtubules decreased (Fig. S4E). This was accompanied by a small but reproducible increase in total Cep152 levels as shown by immunoblotting (Fig. 3B). These observations prompted us to investigate whether Cep152 is not only responsible for targeting the APC/C to the centrosome but is also a substrate of the APC/C. Sequence analysis (Jehl et al., 2016) of Cep152 revealed a potential D box and KEN box pair, the degron recognition motifs of APC/C substrates (Fig. S5B). The D box is conserved in mammals and is present between residues 716 and 742 of human Cep152. Due to the instability of recombinant Cep152, we were unable to both assess direct binding to the APC/C and assay APC/C-mediated ubiquitylation of recombinant Cep152 *in vitro*. We therefore mutated the putative D box and KEN box together (DBK mutant: eGFP–Cep152^DBK^) and created cell lines that inducibly express eGFP-tagged variants of Cep152. Expression of the eGFP-tagged proteins was titrated by the addition of tetracycline. A concentration of 200 pg/ml tetracycline showed similar expression of both transgenes, similar to endogenous protein levels, and was chosen for all subsequent experiments (Fig. S5C). To assess *in vivo* ubiquitylation of Cep152, His-tagged ubiquitin was co-expressed in the cell lines harbouring either wild-type Cep152 (eGFP–Cep152^WT^) or the DBK mutant. Pulldown of His-tagged ubiquitin in cells treated with the proteasome inhibitor MG132 revealed clear evidence for ubiquitylated eGFP–Cep152^WT^ (Fig. 3C). Ubiquitylation of eGFP–Cep152^WT^ was reduced by the small APC/C inhibitors TAME and APCin (Sackton et al., 2014; Zeng et al., 2010) (Fig. 3C), demonstrating that Cep152 ubiquitylation is APC/C dependent. Compared with eGFP–Cep152^WT^, ubiquitylation of eGFP–Cep152^DBK^ was reduced (Fig. 3C). A small amount of ubiquitylation was still visible in the presence of APC/C inhibitors (Fig. 3C). Thus, we cannot exclude the possibility that in this assay additional ubiquitin ligases also target Cep152. However, these results indicate that in cells Cep152 is mainly targeted by the APC/C for ubiquitylation. To further investigate the ubiquitylation of Cep152 by the APC/C, we immunoprecipitated eGFP-tagged Cep152 from HEK cell lines and used these proteins in an *in vitro* ubiquitylation assay with purified APC/C. A portion of eGFP–Cep152^WT^ was shifted to a higher molecular weight species upon incubation with the APC/C. This Cep152 modification depended on the activity of the APC/C and on the presence of the D box and KEN box, being mostly ablated in the eGFP–Cep152^DBK^ mutant (Fig. 3D), consistent with APC/C-catalysed Cep152 ubiquitylation and the results from *in vivo* ubiquitylated Cep152 (Fig. 3C). Incubating the reaction without ubiquitin or with addition of ubiquitin-specific protease 21 (Usp21) also abolished the shift of Cep152, strongly indicating that Cep152 is indeed ubiquitylated and not alternatively modified by the APC/C (Fig. S5D). We noticed that Cep152 ubiquitylation *in vitro* was not very efficient compared to that of other known APC/C substrates (Jin et al., 2008; Zhang et al., 2019). However, our observed ubiquitylation of Cep152 was similar to that of other large APC/C substrates such as Aurora A, MIWI (also known as PIWIL1), p190 (ARHGAP35) and MCPH1, where ubiquitylation often results in a smear rather than a defined ubiquitin ladder (Liu et al., 2017; Min et al., 2015; Naoe et al., 2010; Zhao et al., 2013). To further investigate whether Cep152 is an APC/C substrate, a small peptide that comprises the sequence of Cep152 incorporating the D box and KEN box pair was tested in a ubiquitylation-competition assay. Ubiquitylation of cyclin A2 was abolished in the presence of a peptide modelled on the well-established APC/C substrate Hsl1, as well as when the Cep152 peptide was used (Fig. S5E). A DBK-mutant peptide was insoluble; however, a scrambled control peptide did not compete with the ubiquitylation of cyclin A2. These data support the idea that Cep152 is an APC/C substrate, thereby explaining why levels of Cep152 increase when the APC/C is either depleted or inactivated (Fig. 3A,B). Interestingly, as shown by co-immunoprecipitation, mutation of the Cep152 D box and KEN box does not abolish the binding of Cep152 to the APC/C (Fig. 3E), indicating that the interaction of the APC/C with Cep152 is independent of Cep152 being targeted by the APC/C for ubiquitylation.

### Stabilized Cep152^DBK^ remains at the centrosome during mitosis

Using siRNA-mediated depletion of the endogenous Cep152 protein, we tested whether our eGFP-tagged Cep152 variants are functionally active in the cell and support the established function of Cep152 during centriole duplication (Blachon et al., 2008). In the absence of Cep152, centrioles were not duplicated, and cells showed a reduced number of centrin-3 dots. Both eGFP-tagged Cep152 versions (WT and DBK) were able to rescue the absence of the endogenous protein (Fig. S5F) and restore the expected centriole number of four in mitosis. This is in agreement with previous publications that have found N-terminally-tagged Cep152 to be fully functional (Cizmecioglu et al., 2010; Firat-Karalar et al., 2014; Hatch et al., 2010). We then analysed the localization of eGFP–Cep152 during different mitotic stages. EGFP–Cep152^WT^ served as a control, and we observed that its levels at centrosomes were progressively reduced as cells progressed from early prometaphase to metaphase (Fig. 3F). This behaviour was similar to that of the endogenous wild-type protein (Fig. S5A) and could also explain why depletion of the APC/C (Fig. 3A) only resulted in a small increase in Cep152 at the centrosome in early prometaphase, i.e. Cep152 localization is maximal in early prometaphase and cannot increase further. Notably, compared with levels of eGFP–Cep152^WT^, the centrosomal levels of eGFP–Cep152^DBK^ decreased less markedly as cells progressed through mitosis (Fig. 3F).

### Cep152 is an inhibitor of mitotic spindle assembly

**Fig. 4.**
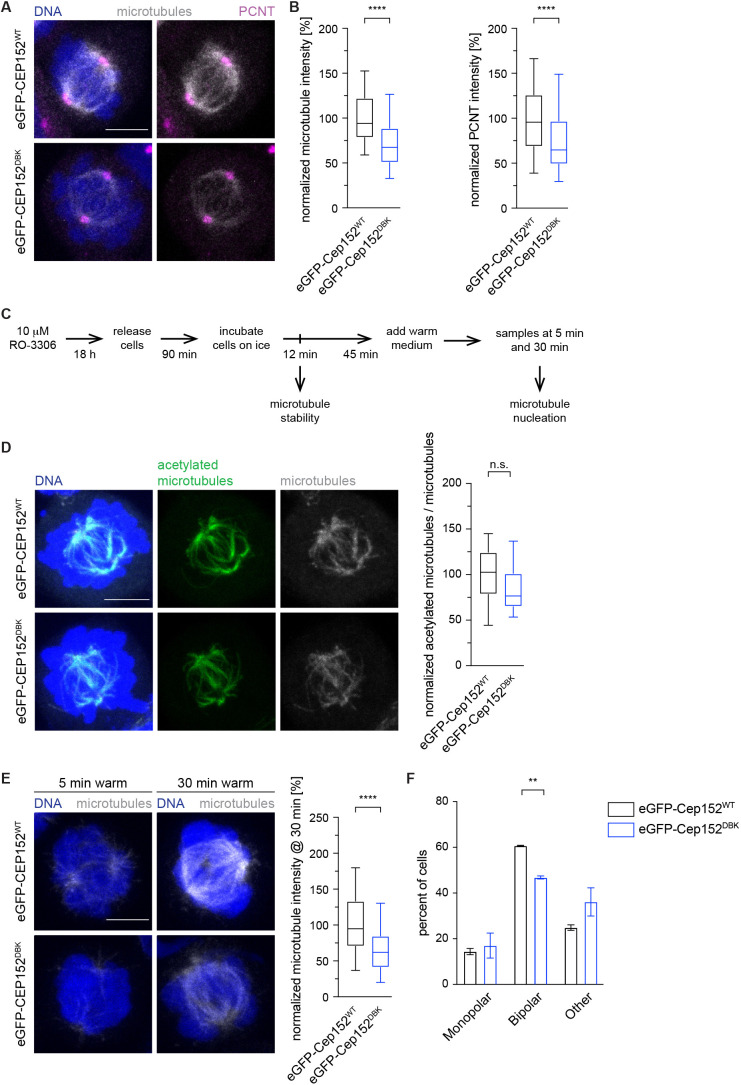
**Cep152 is an inhibitor of mitotic spindle assembly and forms a complex with Cep57.** (A) HEK293 FlpIn T-Rex cell lines expressing eGFP–Cep152^WT^ (WT) or eGFP–Cep152^DBK^ (DBK) were treated as shown in Fig. 2A and stained against the indicated proteins. Microtubules were labelled using an α-tubulin antibody and DNA was stained with DAPI. (B) The fluorescence intensity of tubulin in cells from A was measured in the whole cell and normalized against the eGFP–Cep152^WT^ cell line (left). *N* cells=78 (WT), 77 (DBK). The fluorescence intensity of PCNT at the centrosome in cells from A was measured as indicated in Fig. 1B and normalized against the eGFP–Cep152^WT^ cell line (right). *N* cells=158 (WT), 154 (DBK). ****P<0.0001 (simple one-way ANOVA with Dunnett's multiple comparison test). (C) Setup of experiments to understand microtubule stability and nucleation. Cells were collected at the indicated time points. (D) HEK293 FlpIn T-Rex cell lines expressing eGFP–Cep152^WT^ or eGFP–Cep152^DBK^ were treated according to the schedule in C and fixed to assess microtubule stability. Cells were stained to detect microtubules (anti-α-tubulin) and acetylated microtubules (antibody against acetylated Lys40 of α-tubulin), and DNA was labelled with DAPI (left). The fluorescence intensity of acetylated microtubules was measured in the whole cell and normalized to unmodified microtubules (right). *N* cells=19 (WT) and 23 (DBK). n.s., not significant (Mann–Whitney *U*-test). (E) HEK293 FlpIn T-Rex cell lines expressing eGFP–Cep152^WT^ or eGFP–Cep152^DBK^ were treated according to the schedule in C and fixed to assess microtubule nucleation. Cells were fixed and stained against tubulin and DNA was labelled with DAPI (left). The fluorescence intensity of tubulin was measured in the whole cell at 30 min after shifting cells back to 37°C and normalized against the eGFP–Cep152^WT^ cell line (right). *N* cells=61 (WT), 66 (DBK). ****P<0.0001 (simple one-way ANOVA with Dunnett's multiple comparison test). (F) Cells from E were analysed for their spindle morphology. Data are the mean±s.d. percentages of cells with each spindle phenotype from three experiments. **P=0.0038 (two-tailed paired Student's *t*-test). Box plots in B,D,E show the median (line), 25–75% range (box) and 5–95% range (whiskers). Scale bars: 5 µm.

Having established that Cep152 is responsible for recruiting the APC/C to the centrosome and is itself a substrate of the APC/C, we next investigated the consequences of prolonged Cep152 presence in the cell during mitosis. A mitotic role for Cep152 has not previously been established. We observed that eGFP–Cep152^DBK^ remained at the centrosome for a prolonged time during mitosis (Fig. 3F) and noticed that cells exhibited a strong reduction in spindle microtubule intensity compared to cells expressing eGFP–Cep152^WT^ (Fig. 4A,B), reminiscent of cells where the APC/C was depleted (Fig. S4E). We noticed that the signal intensity of the microtubule nucleators PCNT, and to a lesser extent Cdk5rap2 and γTub, were reduced in the presence of eGFP–Cep152^DBK^, which could explain why fewer microtubules were present (Fig. 4A,B; Fig. S6A). To further investigate this observation, we designed an experiment to distinguish between two possibilities: is either microtubule stability or nucleation altered in cells expressing eGFP–Cep152^DBK^ (Fig. 4C)? When cells were incubated at 4°C for a short time to depolymerize all non-kinetochore (unstable) microtubules, no significant difference was observed between cells expressing either eGFP–Cep152 variant (Fig. 4D). This argues that microtubules in eGFP–Cep152^DBK^-expressing cells are at least partially attached to kinetochores, and therefore a mitotic spindle is formed. To investigate microtubule nucleation, cells were incubated on ice for a longer time to depolymerize stable microtubules. Subsequently, cells were shifted back to 37°C, and nucleation of new microtubules from the centrosome was measured at different time points (Fig. 4E). Under this condition, striking differences were observed between cells expressing the eGFP–Cep152 variants. In cells expressing eGFP–Cep152^WT^, nucleation of new microtubules was observed after 5 min and a bipolar spindle was formed after 30 min (Fig. 4F). In contrast, cells expressing eGFP–Cep152^DBK^ showed a delay in both microtubule nucleation (Fig. 4E) and establishing a bipolar spindle (Fig. 4F). These data support the idea that the proper removal of Cep152 from the centrosome by the APC/C is a critical step for microtubule nucleation. However, once microtubules are attached to kinetochores, they are stable regardless of the presence of remaining Cep152 at the centrosome.

### Cep152 forms a complex with Cep63 to inhibit Cep57

**Fig. 5.**
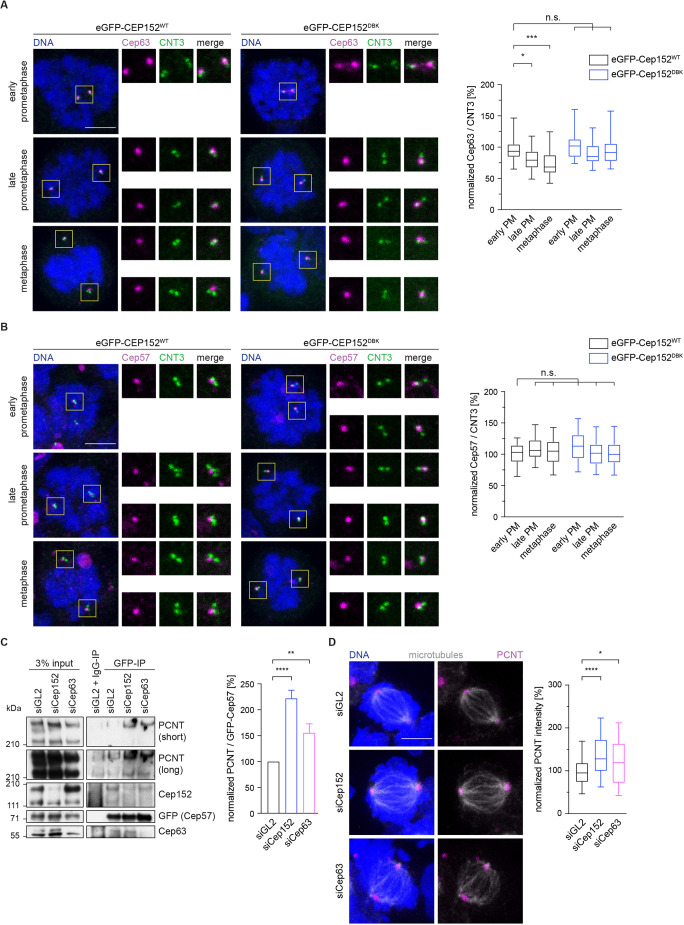
**Persistent Cep152 at the centrosome inhibits PCNT recruitment by Cep57.** (A) HEK293 FlpIn T-Rex cell lines expressing eGFP–Cep152^WT^ (WT) or eGFP–Cep152^DBK^ (DBK) were treated as shown in Fig. 2A and stained with the indicated proteins (left; CNT3, centrin-3). DNA was labelled with DAPI. Boxes indicate regions shown in enlarged images. The fluorescence intensity of Cep63 was measured around the centrosome as indicated in Fig. 1B and normalized against early prometaphase of the eGFP–Cep152^WT^ cell line (right). PM, prometaphase. *N* cells=32 (early PM, WT), 56 (late PM, WT), 27 (metaphase, WT), 32 (early PM, DBK), 54 (late PM, DBK), 33 (metaphase, DBK). *P=0.018; ***P=0.0006; n.s., not significant (simple one-way ANOVA with Dunnett's multiple comparison test). (B) HEK293 FlpIn T-Rex cell lines expressing eGFP–Cep152^WT^ or eGFP–Cep152^DBK^ were treated as shown in Fig. 2A and stained with the indicated proteins (left). DNA was labelled with DAPI. Boxes indicate regions shown in enlarged images. The fluorescence intensity of Cep57 was measured around the centrosome as indicated in Fig. 1B and normalized against early prometaphase of the eGFP–Cep152^WT^ cell line (right). *N* cells=23 (early PM, WT), 48 (late PM, WT), 33 (metaphase, WT), 26 (early PM, DBK), 59 (late PM, DBK), 26 (metaphase, DBK). n.s., not significant (simple one-way ANOVA with Dunnett's multiple comparison test). (C) HEK293 cells expressing eGFP-tagged Cep57 were treated with control siRNA (siGL2), siRNA against Cep152 (siCep152) or siRNA against Cep63 (siCep63) as described in Fig. 2B and were lysed for anti-GFP immunoprecipitation (IP) or control IgG IP. The eluates were probed for the indicated proteins by immunoblotting (left). The PCNT signal in the GFP IP was measured and normalized against the corresponding eGFP–Cep57 signal (right). Mean±s.d. *N* immunoblots=3. **P=0.0036; ****P<0.0001 (one-way ANOVA with Dunnett's multiple comparison test). (D) HEK293 cells were treated with control siRNA (siGL2), siRNA against Cep152 (siCep152), or siRNA against Cep63 (siCep63) as described in Fig. 2B. The cells were fixed and stained against the indicated proteins (left). Microtubules were labelled using an α-tubulin antibody and DNA was stained with DAPI. The fluorescence intensity of PCNT around the centrosome was measured as indicated in Fig. 1B and normalized to the siGL2 control (right). *N* cells=96 (siGL2), 76 (siCep152), 42 (siCep63). *P=0.018; ***P<0.0001 (simple one-way ANOVA with Dunnett's multiple comparison test). Box plots in A,B,D show the median (line), 25–75% range (box) and 5–95% range (whiskers). Scale bars: 5 µm.

Since Cep152 is not known to directly interact with or nucleate microtubules, we speculated that additional interaction partners of Cep152 might be involved in this process. Cep152 forms a stable complex with two other centrosomal proteins, Cep57 and Cep63 (Lukinavičius et al., 2013; Watanabe et al., 2019). In this heterotrimeric complex, Cep63 bridges the interaction between Cep57 and Cep152 (Wei et al., 2020; Zhao et al., 2020). Nucleation of microtubules in mitosis depends on the successful accumulation and expansion of the PCM, comprising proteins such as PCNT and Cdk5rap2 (Gould and Borisy, 1977; Woodruff et al., 2017). Since Cep57 recruits PCNT to centrosomes (Watanabe et al., 2019), we tested whether Cep152 controls centrosomal PCNT levels. Notably, we found that the levels of PCNT at centrosomes were reduced in the presence of stabilized eGFP–Cep152^DBK^ (Fig. 4A,B). We therefore speculated that during mitosis, release of Cep57 from the Cep152–Cep63–Cep57 complex allows it to bind and recruit PCNT. In cells expressing eGFP–Cep152^WT^, we found that levels of both Cep152 and Cep63 decreased during mitosis (Fig. 3F; Fig. 5A). In contrast to this, Cep57 levels at the centrosome remained constant throughout mitosis (Fig. 5B). In agreement with our idea that stabilized Cep152 remains at the centrosome during mitosis and stays bound to Cep63–Cep57, the level of Cep63 was not reduced in cells expressing eGFP–Cep152^DBK^ (Fig. 5A).

These findings suggest the possibility that once levels of Cep152 and Cep63 are reduced at the centrosome, Cep57 is free to recruit additional PCNT to enhance microtubule nucleation. To test our hypothesis directly, we separately depleted Cep152 and Cep63 from cells and performed an immunoprecipitation of eGFP-tagged Cep57 from mitotic cells (Fig. 5C). The immunoprecipitation experiment confirmed previous data showing that Cep63 bridges the interaction of Cep152 with Cep57 (Watanabe et al., 2019; Wei et al., 2020; Zhao et al., 2020), since in the absence of Cep63 less Cep152 was bound to eGFP–Cep57. In contrast, depletion of Cep152 did not reduce the binding of Cep63 to eGFP–Cep57 (Fig. 5C). Cep63 is difficult to deplete in HEK cells: ∼50% of the protein remained even after 96 h of siRNA treatment. Nevertheless, upon siRNA-mediated reduction of either Cep152 or Cep63 more PCNT was associated with eGFP–Cep57 (Fig. 5C), consistent with the hypothesis that the Cep152–Cep63 complex inhibits Cep57–PCNT interactions. Based on this observation, we asked whether more PCNT can be recruited to the centrosome in the absence of Cep152 or Cep63. Performing siRNA-mediated depletion of either protein and subsequent immunofluorescence showed indeed that additional PCNT was present at the centrosome when one of these two proteins was absent (Fig. 5D), confirming our immunoprecipitation data. In conclusion, the removal of Cep152 from the centrosome by the APC/C during mitosis is a critical step to liberate Cep57 from its inhibitory complex and aid faithful microtubule nucleation.

### Stabilized Cep152^DBK^ at the centrosome leads to mitotic errors

**Fig. 6.**
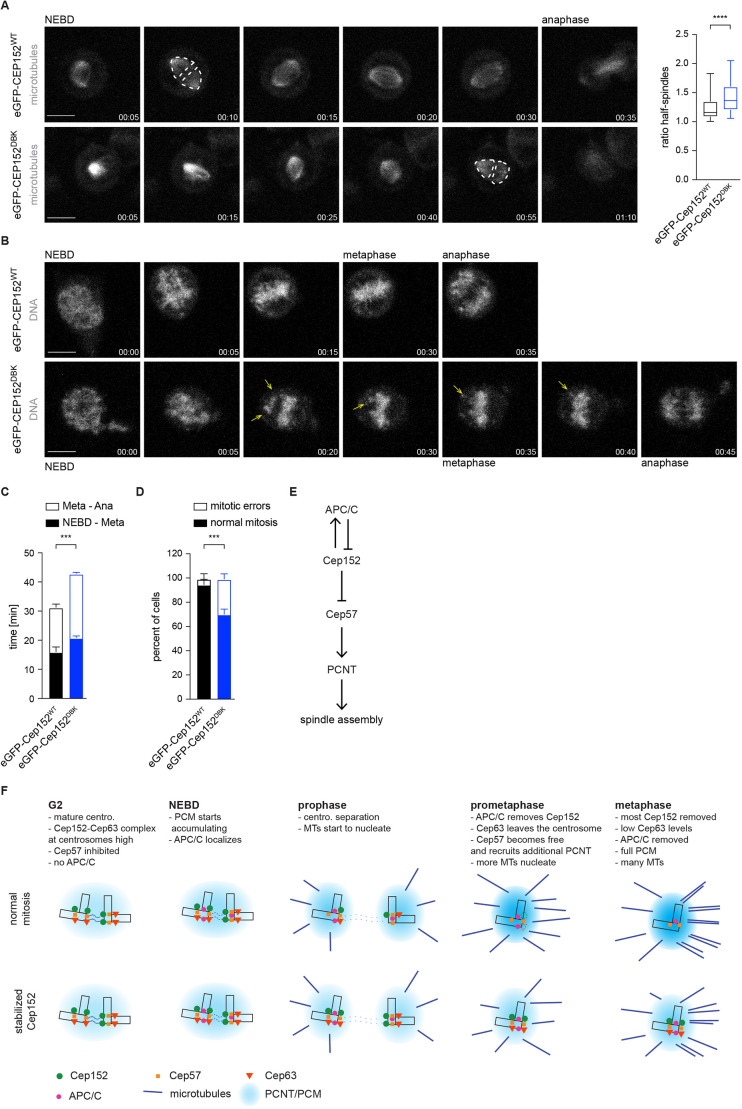
**Persistent Cep152 at the centrosome leads to mitotic errors.** (A) eGFP–Cep152^WT^ (WT) and eGFP–Cep152^DBK^ (DBK) HEK293 cell lines were subjected to live-cell imaging (time is shown as hh:mm). Exemplary still images from the movies are shown. See Movies 5 and 6. Nuclear envelope breakdown (NEBD) and anaphase onset are marked. Microtubules were stained with SiR-tubulin (left). Microtubule intensity was measured in two separate areas (indicated by the dotted lines), called half-spindles, and the brighter area was divided by the dimmer one (right). Every time point was measured in the same way. Box plot shows the median (line), 25–75% range (box) and 5–95% range (whiskers). *N* time points=46 (WT), 56 (DBK). *P=0.006 (Mann–Whitney *U*-test). (B) eGFP–Cep152^WT^ and eGFP–Cep152^DBK^ cell lines were subjected to live-cell imaging (time is shown as hh:mm). Exemplary still images from the movies are shown. See Movies 7 and 8. NEBD, metaphase and anaphase onset are marked. DNA was stained with SiR-DNA. Unaligned chromosomes are indicated by yellow arrows. (C) Cells from B were analysed for the time taken to progress from NEBD to metaphase (NEBD–Meta) and from metaphase to anaphase onset (Meta–Ana). Bar graphs show the mean±s.e.m.. *N*=4 experiments; WT=197 cells, DBK=189 cells. ***P=0.0006 (simple one-way ANOVA with Dunnett's multiple comparison test). (D) Cells from C were analysed for misaligned chromosomes during metaphase and lagging chromosomes during anaphase, combined as mitotic errors. Bar graphs show the mean±s.e.m. *N*=4 experiments. ***P=0.0005 (Holm–Sidak *t*-test). (E) Regulatory network of the APC/C and centrosomal proteins that regulate mitotic spindle assembly. The main APC/C-interacting protein at the centrosome is Cep152, which is also an APC/C substrate, creating a negative feedback loop. (F) Model of microtubule nucleation in the presence of wild-type (top) or stabilized (bottom) Cep152. Centro., centrosome; MTs, microtubules. Scale bars: 10 μm.

Based on our data showing that that microtubule nucleation and formation of a bipolar spindle are slow in the presence of eGFP–Cep152^DBK^ (Fig. 4E,F), we performed live-cell imaging using SiR-tubulin (Lukinavičius et al., 2014) to confirm these results. As expected, nucleation of a mitotic spindle took much longer in cells expressing eGFP–Cep152^DBK^ (Fig. 6A). Furthermore, we noticed that one spindle half was frequently much weaker in its fluorescence intensity compared to the other half. This observation was specific for eGFP–Cep152^DBK^ because in cells expressing eGFP–Cep152^WT^ both half-spindles were equal in their intensity (Fig. 6A; Movies 5,6). Based on this, we reasoned that cells expressing eGFP–Cep152^DBK^ would need more time to complete mitosis. We performed live-cell microscopy imaging of cells expressing the different eGFP–Cep152 variants and analysed the time from nuclear envelope breakdown (NEBD) to metaphase and the time from metaphase to anaphase onset using SiR-DNA (Lukinavičius et al., 2014). In cells expressing eGFP–Cep152^DBK^, both phases were prolonged compared to those in cells with eGFP–Cep152^WT^ (Fig. 6B,C), consistent with our data showing that microtubule nucleation in these cells is diminished (Fig. 4E,F and Fig. 6A). Taken together, these observations could indicate that in the presence of eGFP–Cep152^DBK^, cells have problems correctly attaching their chromosomes to the mitotic spindle, resulting in mitotic errors. Consistent with this, we found that the number of cells that showed either misaligned chromosomes during metaphase or lagging chromosomes during anaphase (together referred to as ‘mitotic errors’) was increased with expression of eGFP–Cep152^DBK^ (Fig. 6D; Movies 7,8). These data highlight the crucial role of APC/C-mediated Cep152 degradation during mitosis, which is required for successful spindle assembly.

## DISCUSSION

This study reveals the crucial role of the APC/C at the centrosome and its function in mitotic spindle assembly. We confirm previous data that the APC/C is part of the mitotic centrosome using co-immunofluorescence as well as co-elution with purified centrosomes (Ban et al., 2007; Meghini et al., 2016; Tugendreich et al., 1995) and show that recruitment of the APC/C to the centrosome depends on Cep152. Interestingly, depletion of the APC/C increases Cep152 levels at the centrosome in mitosis, and we show that Cep152 is itself an APC/C substrate. This negative feedback loop ensures that Cep152 auto-regulates its removal from the centrosome, a step that is important for proper microtubule nucleation in mitosis. Persistent Cep152 at the centrosome binds Cep57 in an inhibitory complex with Cep63 and hinders the recruitment of PCNT, an important microtubule nucleator (Fig. 6E,F).

Our results do not exclude that there are additional APC/C substrates and interacting proteins localized to the centrosome. For example, the kinase Nek2A predominantly localizes to the centrosome in early mitosis until it is degraded by the APC/C (Faragher and Fry, 2003; Fry et al., 1998). Recently, it has been shown that the *Drosophila* homologue of Cep192 (Spd2) recruits the APC/C co-activator Cdh1 to the centrosome and that Cep192/Spd2 is also an APC/C substrate (Meghini et al., 2016; Raff et al., 2002). This observation is supported by our data, as we found Cdh1 co-eluting with isolated centrosomes and detected Cep192 in our APC/C proximity labelling. It is therefore possible that other proteins like Cep192 or Cep131 are also involved in recruiting the APC/C to the centrosome. An intriguing idea would be that different parts of the APC/C, such as the core complex and the co-activators, are localized to the centrosome by separate processes, with a functional complex only being assembled when needed. This could favour a rapid activation of the APC/C at the centrosome to ensure that Cep152 is targeted early in mitosis, because persistent Cep152 delays microtubule nucleation. The details of the mechanisms responsible for regulating APC/C activity localized to centrosomes are not currently understood.

A function of Cep152 in mitotic spindle assembly has not been previously established, probably because a stabilized Cep152 mutant has not been available. Our work, therefore, also implicates Cep152 as an inhibitor of mitotic spindle formation. We postulate that to ensure the timely removal of Cep152 from the centrosome, it auto-regulates its local ubiquitylation by the APC/C. This very elegant dual function of Cep152 as a recruiter as well as a substrate of the APC/C ensures that Cep152 is ubiquitylated at centrosomes in a local and timely fashion, and not throughout the cell. We show that Cep152 accumulates only slightly throughout the cell in the absence of APC/C activity. This suggests that ubiquitylation of Cep152 by the APC/C could result in relocation of Cep152 from the centrosome, but not necessarily in its degradation. This behaviour has not been reported previously for APC/C substrates but is known in other contexts (Huang et al., 2003; Kruse and Gu, 2009; Yao et al., 2018).

We show that the APC/C localizes to the centrosome inside the PCM. This behaviour is similar to other proteins such as PCNT, Cdk5rap2 and γTub, which, like the APC/C, reside in a similar layer surrounding the centrosome (Lawo et al., 2012; Sonnen et al., 2012). The main interacting protein of the APC/C at the centrosome is Cep152; however, we cannot formally exclude that other proteins, such as Cep131 or Cep192, could also contribute the APC/C localizing to the centrosome. Cep152 in its heterotrimeric complex with Cep63 and Cep57 has previously been shown to localize to the proximal end of the centriole, forming a ring-like pattern (Ito et al., 2021; Lukinavičius et al., 2013; Sonnen et al., 2012; Zhao et al., 2020). This is in agreement with our data showing such localization for the APC/C. Our data establish that in the absence of Cep152, the APC/C can no longer localize properly to the centrosome. This localization, however, is important, as interfering with the timely removal of Cep152 from centrosomes by the APC/C results in reduced microtubule nucleation and consequently in chromosome alignment defects and segregation errors. These phenotypes are very similar to those observed following Cep57 depletion (Watanabe et al., 2019; Wu et al., 2012), and we established that the Cep152-Cep63 complex has an inhibitory function on Cep57. In the presence of stabilized Cep152, Cep63 remains at the centrosome and Cep57 is not released from this inhibition, which hampers the recruitment of PCNT.

In a recent proteomic study, the APC/C has been identified as a minor component of centriolar satellites (Gheiratmand et al., 2019). This suggests the possibility that the APC/C could be a dynamic component of centriolar satellites before mitosis and could be transported to the centrosome together with some of its interacting proteins, for example Cep131 and Cep170 (Barenz et al., 2018; Gheiratmand et al., 2019; Gupta et al., 2015; Quarantotti et al., 2019). If satellite-localized Cep170 is involved in transporting the APC/C to the centrosome, this could explain our results that Cep170 depletion leads to a failure in APC/C localization to the centrosome. A similar effect could also contribute to the Cep131-depletion phenotype, where the APC/C is also absent from centrosome. However, in this instance a direct relationship between Cep131 and Cep152 has previously been established (Kodani et al., 2015) and is confirmed by our study, and we propose that this accounts for the main phenotype. Nevertheless, the interaction of the APC/C with centriolar satellites remains to be investigated in the future.

Taken together, our study establishes how the APC/C is recruited to the centrosome and reveals its critical function in local ubiquitylation of Cep152, an inhibitor of mitotic spindle assembly. It also shows that the APC/C has a dual function during mitosis, where it is not only involved in mitotic progression, but also directly regulates spindle assembly.

## MATERIALS AND METHODS

### Tissue culture and cell cycle synchronization

HEK293 FlpIn-TRex cells (Invitrogen, USA) were cultured in DMEM (Gibco, USA) supplemented with 10% tetracycline-free FBS (PAN Biotech, Germany) at 37°C and 5% CO_2_. RPE1 cells (a gift from Dean Clift, MRC Laboratory of Molecular Biology, Cambridge, UK) were cultured in DMEM:F12 (Gibco, USA) supplemented with normal FBS (PAN Biotech, Germany) at 37°C and 5% CO_2_. Cell lines were tested for mycoplasma contamination before aliquots were frozen for storage. For the stable integration of eGFP–Cep131, eGFP–Cep192, eGFP–Cep152 (WT and DBK), eGFP–APC2 and APC3–eGFP into the genome, the corresponding genes were cloned into the pcDNA5-FRT-TO vector (Invitrogen, USA) with an N-terminal or C-terminal (only in the case of APC3) eGFP tag. HEK293 FlpIn-TREX cells were co-transfected with the respective pCDNA5-FRT-TO plasmid and pOG44 (Invitrogen), containing the flp-recombinase, using HBS buffer. Briefly, cells were seeded the evening before transfection, and the medium was exchanged the next morning. Both plasmids were mixed with 160 mM CaCl_2_ and 2× HBS buffer (final concentrations: 137 mM NaCl, 5 mM KCl, 0.7 mM Na_2_HPO_4_, 7.5 mM D-glucose, 21 mM HEPES) and added to the cells. The next steps were performed according to the Invitrogen manual. Cells were selected using 100 µg/ml hygromycin B gold (InVivoGen, USA). For creation of the BioID2–APC2 and APC3–BioID2 cell lines the same protocol was performed, but the pcDNA5-FRT-TO vector was modified to either include an N-terminal or a C-terminal BioID2 tag. All stable cell lines were kept under selection. Protein expression was induced with 200 pg/ml doxycycline (Sigma, USA). Cell cycle synchronization was performed using a combination of 2.5 mM thymidine (Sigma) to arrest cells in or before S phase and either 300 nM nocodazole (Sigma, USA), 500 nM taxol (Sigma, USA), 5 µM STLC (Sigma, USA) or 10 µM RO-3305 (Santa Cruz, USA) for a mitotic arrest. Cells were released from thymidine after 16 h by washing three times with pre-warmed medium using the same volume as during culture. The second drug was added 4 h after thymidine release and incubated for another 16 h. Release from RO-3306 was performed in the same way. Depletion of centrosomes was achieved by treatment of cells with 150 nM centrinone (Sigma, USA) for 7 days.

### RNAi-mediated protein depletion

Cells were seeded at a density of 50% 16 h before transfection with siRNA. RNAi was performed using RNAiMAX reagent (Invitrogen, USA) according to the manual. For the depletion of all proteins a double depletion protocol using two times 20 nM oligo was used. In short, 16 h after seeding the cells were transfected with the first round of siRNA, and 8 h later thymidine was added (see above). Again, 16 h later the cells were released from thymidine and the second round of siRNA transfection was performed. The thymidine block and release were repeated, and cells were arrested in mitosis afterwards (see above). This protocol ensures that most of the cells only perform one round of mitosis, which avoids problems with centrosome duplication (in case of the depletion of centrosomal proteins) or cell cycle arrest (in case of APC/C depletion). The following siRNA oligos were used: GL2, 5′-AACGUACGCGGAAUACUUCGA[dT][dT]-3′; APC6_5, 5′-AUGAUGCUCUAGAUAACCGAA[dT][dT]-3′; APC6_6, 5′-CCCAUGCACUUCGGUCACGAA[dT][dT]-3′; APC2_6, 5′-CUCACUGGAUCGUAUCUACAA[dT][dT]-3′; APC2_5, 5′-AAGGUUCUUCUACCGCAUCUA[dT][dT]-3′; Cep131, 5′-CUGACAACUUGGAGAAAUU[dT][dT]-3′; Cep170, 5′-GAAGGAAUCCUCCAAGUCA[dT][dT]-3′; Cep350, 5′-AUGAACGAUAUCAGUGCUAUA[dT][dT]-3′; Cep192, 5′-AAGGAAGACAUUUUCAUCUCU[dT][dT]-3′; Cep152, 5′-GCGGAUCCAACUGGAAAUCUA[dT][dT]-3′; Cep63, 5′-GGCUCUGGCUGAACAAUCA[dT][dT]-3′.

### Immunoblotting

For immunoblotting, ∼3×10^6^ cells/ml were washed once with phosphate-buffered saline (PBS), spun down and resuspended in NuPAGE LDS sample buffer (Invitrogen, USA). After heating the solution for 5 min at 95°C, 5–10 μl were loaded on to a Novex Bis-Tris 4–12% gel (Invitrogen, USA) and run for the appropriate time. The proteins were transferred to a nitrocellulose membrane (GE Healthcare, USA), which was blocked with 5% dried milk in PBS-Tx (PBS containing 0.1% Triton X-100). The primary antibodies were incubated over night at 4°C; the secondary antibodies were used for 1 h at room temperature. The following antibodies were used for immunoblotting: pericentrin (Abcam, ab4448; 1:2000), APC8 (Abcam, ab182003; 1:1000), APC6 (Cell Signaling Technology, 9499; 1:1000), APC3 (Cell Signaling Technology, 12530; 1:1000), APC2 (Cell Signaling Technology, 12301; 1:1000), CDH1 (Abcam, ab89535; 1:1000), BubR1 (Abcam, ab54894; 1:500), Mad2 (Abcam, ab10691; 1:1000), APC3 (Sigma, C7104; 1:500), γ-tubulin (Sigma, T6557; 1:1000), β-actin (Santa Cruz Biotechnology, sc-47778-HRP; 1:2000), α-tubulin (Bio-Rad, MCA78G; 1:2000), Cdc20 (Santa Cruz Biotechnology, sc-8358; 1:100), Bub3 (BD Biosciences, 811730; 1:500), Cep131/AZI-1 (Bethyl Laboratories, A301-415A; 1:1000), Cep170 (Abcam, ab72505; 1:500), Cep350 (Novus Biologicals, NB100-59811; 1:500), Cep152 (Bethyl Laboratories, A302-480A; 1:500), Cep192 (Bethyl Laboratories, A302-324A; 1:1000), cyclin B1 (Abcam, ab72; 1:1000), His (Takara/Clontech, 631212; 1:1000), GFP (Roche, 11814460001; 1:1000), Cep57 (GeneTex, GTX115931; 1:1000), Cep63 (a gift from the Fanni Gergely laboratory, Cancer Research UK, Cambridge, UK), phosphorylated histone H3 (Millipore, 06-570; 1:1000), secondary anti-rabbit (Thermo Fisher Scientific, 31462; 1:10,000), secondary anti-mouse (Agilent, P0260; 1:10,000), secondary anti-rat (Santa Cruz Biotechnology, 2032; 1:10,000).

### Centrosome purification

Mitotic centrosomes were purified from 2×10^8^ HEK293 cells. Cells were synchronized with thymidine and nocodazole (see above). Cytochalasin D (1 µg/ml; Sigma, USA) was added 1 h before harvesting the cells. Mitotic cells were collected by shake off and gentle washing using medium. All subsequent steps were performed at 4°C. Cells were washed once in PBS, once in 0.1× PBS containing 8% sucrose, and once in 8% sucrose in H_2_O. The cells were resuspended in 10 ml lysis buffer [1 mM PIPES, 0.1 mM EGTA, 0.1% 2-mercaptoethanol, 0.5% Triton X-100 and EDTA-free cOmplete protease inhibitors (Roche, Switzerland)] and incubated for 10 min. The lysate was centrifuged at 2500 ***g*** for 10 min, and the supernatant filtered through a 40 µm cell strainer. From a 50× stock of PE (500 mM PIPES, 50 mM EGTA, pH 7.2), the respective volume to prepare a 1× solution was added to the supernatant. Sucrose solutions with 70% (w/v), 60% (w/v), 50% (w/v) and 40% (w/v) were prepared in 1× PE. The cell lysate was carefully layered over 1 ml 60% sucrose cushion in a thin-walled Beckman tube (344058) and centrifuged at 10,000 ***g*** for 30 min using a slow deceleration. About 70% of the tube content was removed from the top and discarded and the remaining volume was mixed with the 60% sucrose cushion to get solution of about 20% sucrose. In parallel, a sucrose gradient consisting of 2 ml 70% sucrose (bottom), 1.2 ml 50% sucrose (middle) and 1.2 ml 40% sucrose (top) was prepared in a Beckman tube (344060). The cell lysate containing 20% sucrose was layered over the sucrose gradient and centrifuged for 16 h at 100,000 ***g***. Deceleration was performed without using the centrifuge brake. The tube with the gradient was pierced at the bottom with an 18G needle, and fractions (F) of the following sizes were collected: F1–F3, 500 µl each; F4– F9, 200 µl each; F10 and F11, 500 µl each; F12 and F13, 1 µl each. Centrosome-containing fractions, as determined by immunoblotting, were snap frozen in liquid nitrogen.

### Mass spectrometry

To prepare samples for mass spectrometry, cells were treated as described above, but were additionally incubated with 50 µM biotin (Sigma, USA) for 24 h before harvesting. After centrosomes were purified, all fractions from the corresponding cell lines were combined and diluted with three volumes of lysis buffer plus 0.1% SDS. The solution was sonified using a microtip with the following settings: 1 min time, 10 s on, 20 s off, 45% power. The sonified centrosomes were incubated with magnetic streptavidin beads (MyOne Streptavidin C1; Thermo Fisher Scientific, USA) overnight. Washing was performed one time each in the following order: lysis buffer, SDS buffer (2% SDS in H_2_O), salt buffer (500 mM NaCl, 1% Triton X-100, 1 mM EDTA and 50 mM HEPES, pH 7.6), Tris buffer (50 mM Tris-HCl, 50 mM NaCl, pH 7.6). Purified proteins were eluted from the streptavidin beads with Tris buffer plus 5 mM biotin. The whole samples were run on a NuPAGE SDS gel and stained with InstantBlue (Expedeon, UK), and each lane was cut into ten equally sized pieces, which were send for mass spectrometric analysis as described previously (Zhang et al., 2016).

### Co-immunoprecipitation

HEK cells were treated with siRNA or compounds for synchronization as described above. For eGFP–Cep152 immunoprecipitation, cells were lysed in the same way as described for the centrosome purification up until the addition of PE buffer. For eGFP–Cep57 immunoprecipitation, cells were lysed in a Tris buffer (20 mM Tris-HCl pH 7.4, 50 mM NaCl, 5 mM EGTA, 2 mM MgCl_2_, 1 mM DTT, 0.5% Triton X-100 and cOmplete protease inhibitors) for 30 min on ice. In both cases, the lysate was centrifuged for 10 min at 2500 ***g*** at 4°C and the supernatant was carefully taken off. Subsequently, it was incubated with 5 µg anti-GFP antibody (Roche, Switzerland) or normal mouse IgG (Santa Cruz, USA) coupled to magnetic Protein G beads (Invitrogen, USA) for 3–4 h at 4°C under constant agitation. The beads were washed three times with the corresponding lysis buffer (with 150 mM NaCl in the case of eGFP–Cep152) and eluted by heating in NuPAGE LDS Sample Buffer (Invitrogen, USA) with 5 mM DTT.

### Live-cell microscopy

An SP8 confocal microscope (Leica, Germany) equipped with a heated environmental chamber, an argon laser, a 630 nm laser line and an APO CS2 40×/1.1 water immersion lens was used for live-cell imaging. Live-cell microscopy was performed as described previously (Zhang et al., 2019). The imaged cells were analysed either for their microtubule intensity with 100 nM SiR-tubulin (Spirochrome, France) or for the time of NEBD, the time when a metaphase plate was observed for the first time and the time of anaphase onset with 250 nM SiR-DNA (Spirochrome, France). Mitotic errors that were visible during the movies were noted and analysed.

### Immunofluorescence microscopy and antibodies

After cells were grown in tissue culture plates, mitotic cells were collected by a gentle wash with medium and spun down onto poly-lysine (Sigma)-treated coverglasses for 5 min at 650 ***g***. Cells were fixed using either methanol or formaldehyde. Except for where noted, cells were pre-extracted for 2 min at 37°C using 0.2% Triton X-100 in PHEM buffer (60 mM PIPES, 25 mM HEPES, 10 mM EGTA, 2 mM MgCl_2_, pH 7.5). For methanol fixation, cells were incubated for 5 min at −20°C with pre-cooled methanol. Afterwards cells were rehydrated with PBS and washed with PBS-Tx, before blocking with ABDIL (1% BSA and 0.1% Triton X-100 in PBS). For formaldehyde fixation, cells were incubated two times for 5 min at 37°C with pre-warmed 3.7% formaldehyde in PHEM buffer. Subsequently cells were washed in PBS-Tx and blocked in ABDIL. Primary antibody incubation was performed for 1 h at room temperature. After washing three times with ABDIL, the secondary antibodies were incubated for 1 h at room temperature, and samples were washed again three times in ABDIL. If microtubules were stained, the process was repeated with an anti-α-tubulin antibody (Bio-Rad, MCA78G; 1:2000) to minimize cross-reaction. In the last washing step, 1 µg/ml DAPI (Sigma) was included. Primary antibodies used for staining were: pericentrin (Abcam, ab4448; 1:2000), APC2 (Cell Signaling Technology, 12301; 1:1000), APC3 (Sigma, C7104; 1:500), γ-tubulin (Sigma, T6557; 1:1000), Cep131/AZI-1 (Bethyl Laboratories, A301-415A; 1:1000), Cep170 (Abcam, ab72505; 1:500), Cep350 (Novus Biologicals, NB100-59811; 1:500), Cep152 (Bethyl Laboratories, A302-480A; 1:500), Cep192 (Bethyl Laboratories, A302-324A; 1:1000), GFP (Abcam, ab6556; 1:1000), centrin-3 (Novus Biologicals, H00001070-M01; 1:1000), Cep57 (GeneTex, GTX115931; 1:500), Cep63 (Millipore, 06-1292; 1:100), Cdk5rap2 (a gift from the Fanni Gergely laboratory; 1:500), Mad2 (Santa Cruz Biotechnology, 65492; 1:50), α-tubulin (Bio-Rad, MCA78G; 1:2000), acetylated tubulin (Sigma, T6792; 1:2000). Secondary antibodies were pre-absorbed and Alexa Fluor coupled, and were purchased from Thermo Fisher Scientific, USA. Alexa Fluor 488-, Alexa Fluor 568- and Alexa Fluor 647-coupled antibodies were used in all combinations against mouse, rabbit or rat primary antibodies at a 1:1000 dilution.

Images were acquired with an SP8 confocal microscope (Leica, Germany) equipped either with 405 nm, 488 nm, 568 nm and 633 nm laser lines, or with a white-light laser and a 592 nm STED laser. Both microscopes were used with an APO CS2 63×/1.4 oil immersion objective. Image resolution was set to 1024×1024 with a 3× or 5× zoom-in, bi-directional scanning, 600 Hz scan speed and 2× line average. A *z*-spacing of 0.23 µm was always used. Laser powers and detector gain were set for each primary antibody individually but were kept constant between different experiments with the same antibody to ensure reproducibility.

### Image quantification and statistical analysis

Fluorescence images were analysed with ImageJ (NIH, Bethesda, MD, USA). Maximum intensity projections were created, and a circular region of interest was used for the measurements. This was either be a small circle of 1–2 µm diameter in the case of centrosomes (see Fig. 1B and Fig. S1C for examples) or a circle encompassing the whole cell (Fig. 1C, Fig. 4C,D; Fig. S1C); the background was measured inside the cells. The integrated density was corrected for the area and the background. All values for the control condition in each experiment were averaged, and this mean value was used to normalize all other conditions against the control using Excel (Microsoft, USA). The normalized values were exported to Prism 9 (Graphpad, USA) and plotted. All boxplots display the 5–95% range (whiskers), the 25–75% range (box) and the median (line). Outliers are not shown, but were included in the statistical analysis. Bar graphs display the mean±s.d. Statistical analysis was performed in Prism 9, and the tests that were used are described in the corresponding figure legends. All experiments were repeated at least twice using biological replicates. The experimenter was not blinded.

### 2D and 3D dSTORM

Direct STORM (dSTORM) was performed with a Nikon N-STORM equipped with 405 nm, 488 nm, 561 nm and 647 nm laser lines (Agilent/Keysight MLC 4008) using an APO TIRF 100×/1.49 oil immersion objective. Cells were cultured in an 8-well glass-bottom slide (Ibidi, Germany) and fixed and stained as described above. The secondary antibodies were pre-coupled to Alexa Fluor 568 or Alexa Fluor 647 (Thermo Fisher Scientific; 1:500) and were additionally fixed with 4% formaldehyde after the staining was concluded (post fix). Before imaging, the wells were incubated for 5 min with Tetraspeck beads (Thermo Fisher Scientific; 1:100) and subsequently washed with PBS. The cells were imaged in switching buffer [100 U glucose oxidase (Sigma, USA), 10,000 U catalase (Sigma, USA), 8% glucose, 100 mM MEA (Sigma, USA), pH 7.6] by filling the well of the Ibidi slide to the top and covering it with a coverglass, avoiding air bubbles. For 3D imaging, a cylindrical lens was inserted into the light path. STORM was performed by inducing and observing blinking events of the Alexa Fluor-coupled secondary antibodies – usually 50,000 frames with 10 ms exposure were recorded. Pre- and post-STORM widefield images were acquired as well. Point fitting in 2D and 3D space was performed within the Nikon software. For 2D-dSTORM, the resulting images were directly saved from the Nikon software. For 3D-dSTORM the fitted point coordinates were exported, transformed in ImageJ using ChriSTORM (Leterrier et al., 2015) and imported using the ThunderSTORM (Ovesný et al., 2014) ImageJ plugin. If necessary, two-colour images were manually aligned using the Tetraspeck beads as reference points with the ‘Align RGB planes’ plugin (https://imagej.net/plugins/align-rgb-planes) in ImageJ. A gaussian normalization algorithm was used for visualization and the data were rendered in 3D using the ImageJ 3D viewer. The distance between two APC/C dots to estimate centrosomal diameter was measured using the ‘Peak finder’ plugin (https://imagej.net/plugins/find-peaks) in ImageJ. The size of APC/C dots around the centrosome and of purified APC/C was measured using a Full Width Half Max (FWHM) macro written by John Soon Yew Lim (A*STAR Skin Research Institute of Singapore) in ImageJ.

### Ubiquitylation assays

*In vivo* ubiquitylation was performed by transfecting HEK293 cells carrying eGFP-tagged Cep152 versions with a plasmid expressing di-ubiquitin-His6× (gift from Leo Kiss, MRC Laboratory of Molecular Biology, Cambridge, UK) for 48 h. For the last 18 h of the expression, cells were treated with 20 µM TAME (Boston Biochem, USA) and 100 µM APCin (Sigma, USA) and/or 20 µM MG132 (Sigma, USA). Cells were harvested and washed once in PBS before lysis in urea buffer (8 M urea, 50 mM Tris-HCl pH8, 10 mM imidazole, 100 mM NaCl) for 30 min at room temperature. The lysate was centrifuged for 10 min at 20,000 ***g***, and the supernatant was incubated for 3 h at room temperature with Ni-NTA beads (Qiagen, Germany) equilibrated in urea buffer. The beads were washed one time each in urea buffer, a 1:1 mix of urea buffer and wash buffer (20 mM Tris-HCl pH 8, 20 mM imidazole, 0.1% Triton X-100, 100 mM NaCl), a 1:3 mix of urea buffer and wash buffer, and finally wash buffer. The beads were eluted with 1× NuPAGE LDS sample buffer containing 300 mM imidazole.

EGFP-tagged Cep152 for the ubiquitylation assay was immunoprecipitated from nocodazole-arrested HEK cells. For this, cells were harvested, washed in PBS and lysed in buffer (150 mM NaCl, 1 mM MgCl_2_, 50 mM HEPES pH 8, 1 mM EGTA, 0.5% Triton X-100 and cOmplete protease inhibitors). The lysate was centrifuged for 10 min at 2500 ***g*** at 4°C, and the supernatant was carefully taken off. It was subsequently incubated with 5 µg GFP antibody (Roche, Switzerland) or normal mouse IgG (Santa Cruz Biotechnology, USA) coupled to magnetic Protein G beads (Invitrogen, USA) for 2 h at 4°C under constant agitation. The beads were washed three times using lysis buffer with additional 150 mM NaCl (for a total 300 mM NaCl) and eluted by incubation with glycine buffer (150 mM NaCl, 200 mM glycine, pH 2.3) for 10 min on ice. The solution was neutralized by addition of a corresponding volume of 1 M HEPES pH 8 to reach pH 7.6.

*In vitro* ubiquitylation was performed using APC/C and Cdc20 purified from insect cells. 60 nM APC/C, 30 nM Cdc20, 90 nM UBA1, 300 nM UbcH10 or 300 nM UbcH5, 35 µM ubiquitin, 5 mM ATP and 10 mM MgCl_2_ were mixed in a buffer containing 40 mM HEPES (pH 8.0), 80 mM NaCl and 0.6 mM DTT. The reaction was mixed with 50% eluted Cep152 and incubated for 60 min at 23°C. The assay was stopped by the addition of one volume of 2× concentrated NuPAGE LDS loading buffer.

## Supplementary Material

Supplementary information

Reviewer comments
