# Supplementary material for: The APC/C targets the Cep152–Cep63 complex at the centrosome to regulate mitotic spindle assembly
Source: J Cell Sci. 2022 Jan 18;135(2):jcs259273. doi: 10.1242/jcs.259273 (PMC8917351; doi:10.1242/jcs.259273)
Supplement: Supplementary information [file joces-135-259273-s1.pdf]

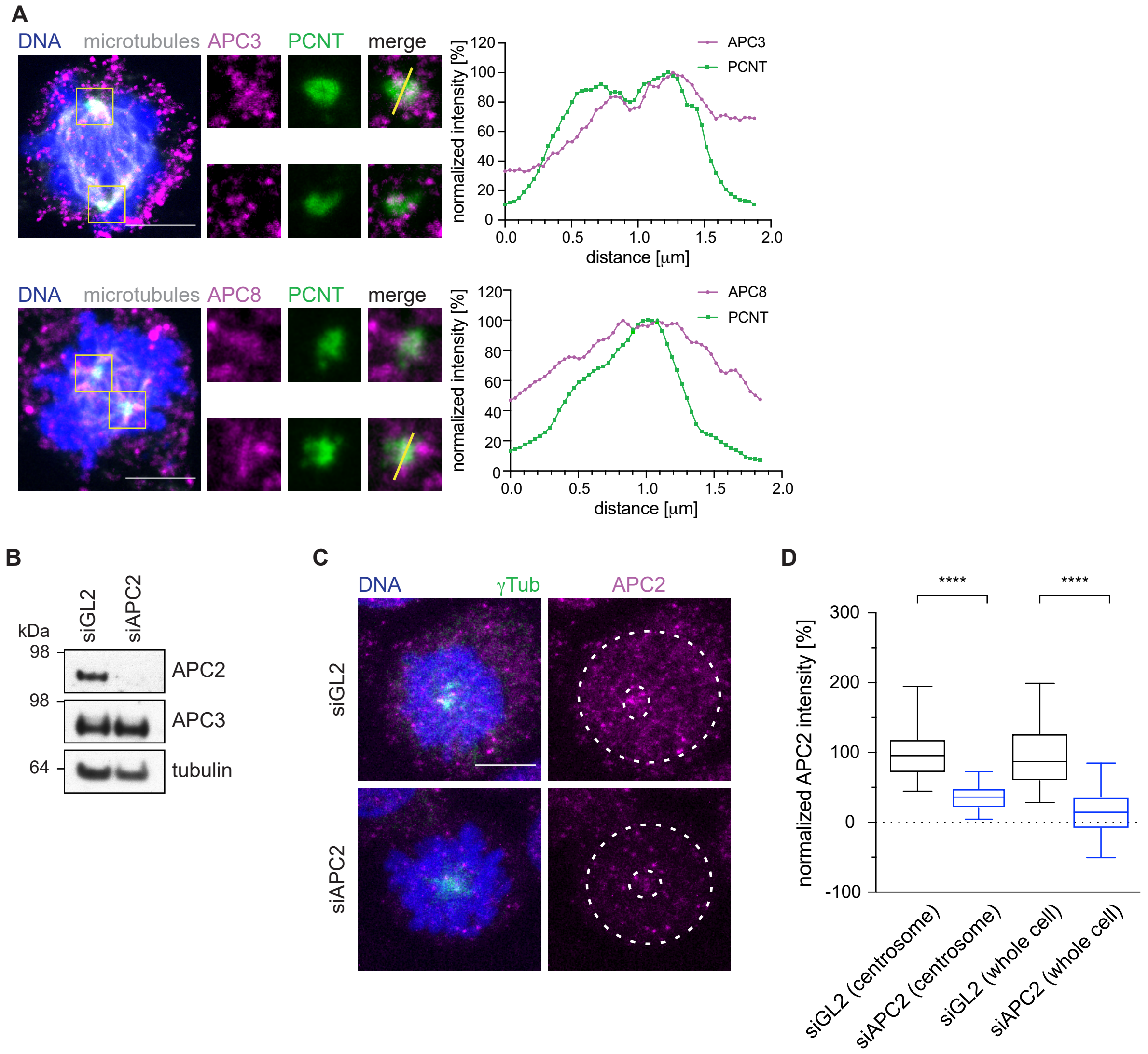

Fig. S1. Specificity of the APC2 antibody used in this study. A, HEK293 cells were fixed with formaldehyde and stained against the indicated APC/C subunits and marker proteins. A line scan with a 10 pixel wide line was performed at the indicated positions and the normalized values were plotted. **B**, Immunoblot from whole cell lysate of cells treated with control siRNA (siGL2) or siRNA against APC2 (siAPC2). **C**, Cells treated as in **B** were fixed in formaldehyde (no pre-extraction) and stained with the indicated antibodies. **D**, quantification from cells shown in **C.** The measurement was either performed around the centrosome as defined by a circle around the γTubulin signal or in the whole cell, as indicated by the dotted lines. All scale bars are 5 µm. ****$P<0.0001$ using a Mann-Whitney U-test. N = number of cells = 107 (siGL2) and 102 (siAPC2).

Journal of Cell Science • Supplementary information

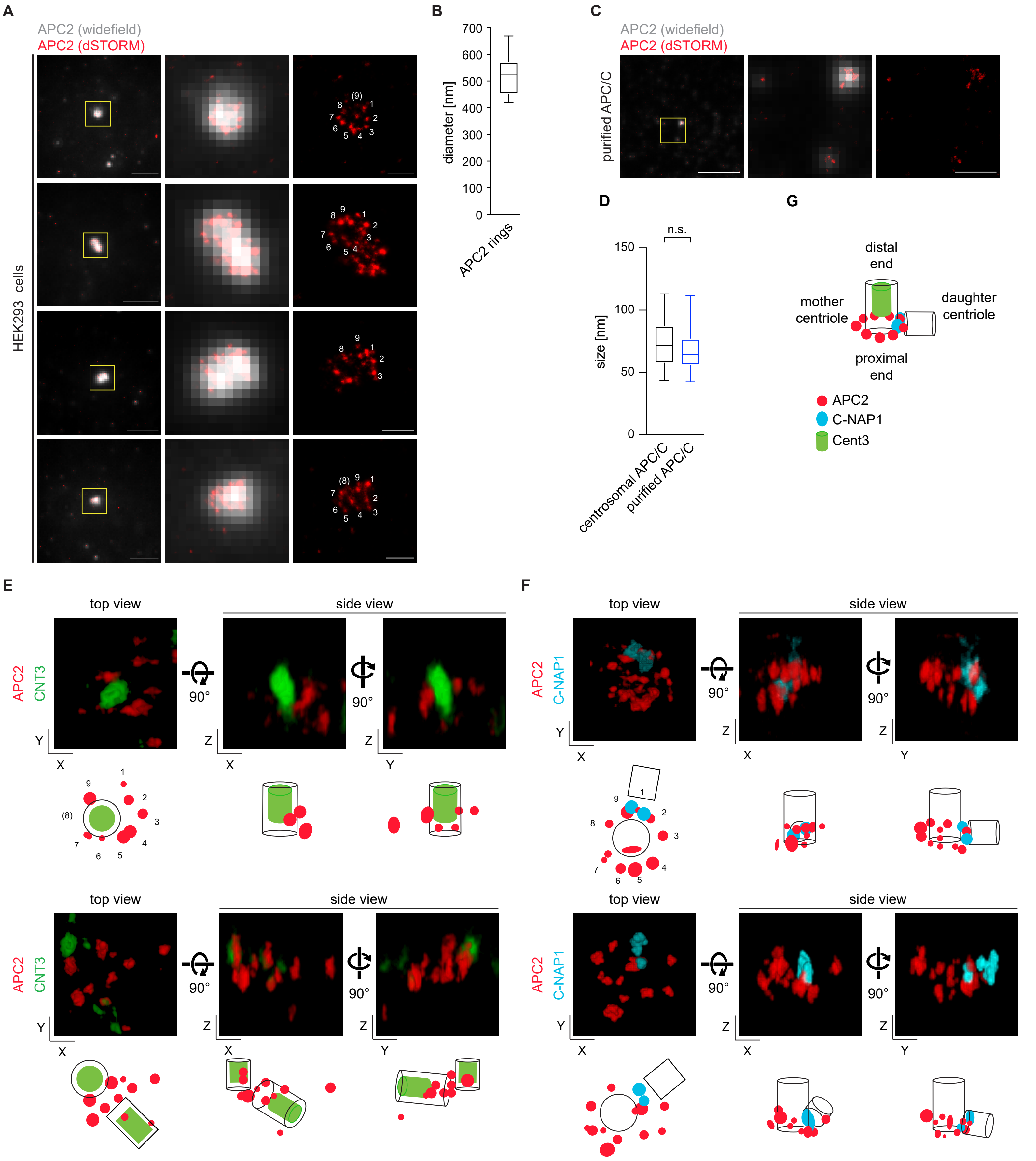

Fig. S2. dSTORM imaging of the APC/C at mitotic centrosomes. A, HEK293 cells were arrested in prophase using taxol, pre-extracted and fixed with formaldehyde. APC2 was stained and dSTORM imaging was performed. The merged image shows the diffraction limited widefield image and the reconstructed dSTORM image. The numbers on the dSTORM image indicate the number of APC/C complexes that can be counted. Scale bars are 5 µm in the overview and 0.5 µm in the inset. **B**, The average diameter of the APC/C rings shown in **A** was measured in at least two directions per centrosome. N = 20. **C**, Purified APC/C was fixed with formaldehyde. APC2 was stained and dSTORM imaging was performed. The merged image shows the diffraction limited widefield image and the reconstructed dSTORM image. Scale bars are 5 µm in the overview and 0.5 µm in the inset. **D**, The average diameter of the signal dots measured after STORM imaging of centrosomal APC/C or purified APC/C. N = 212 (centrosomal APC/C), 101 (purified APC/C). **E**, **F**, Cells were treated as described in **a**, but additionally stained against the indicated proteins. Two-color 3D STORM was performed using a cylindrical lens and by imaging both channels sequentially. Three different views are shown. The illustrations below the images indicate the possible orientation of the centrioles. See also Supplementary movies S1 - S4. **G**, A model of the APC/C localization around the centrosome. The APC/C localizes within the PCM towards the proximal end of the centriole.

Journal of Cell Science • Supplementary information

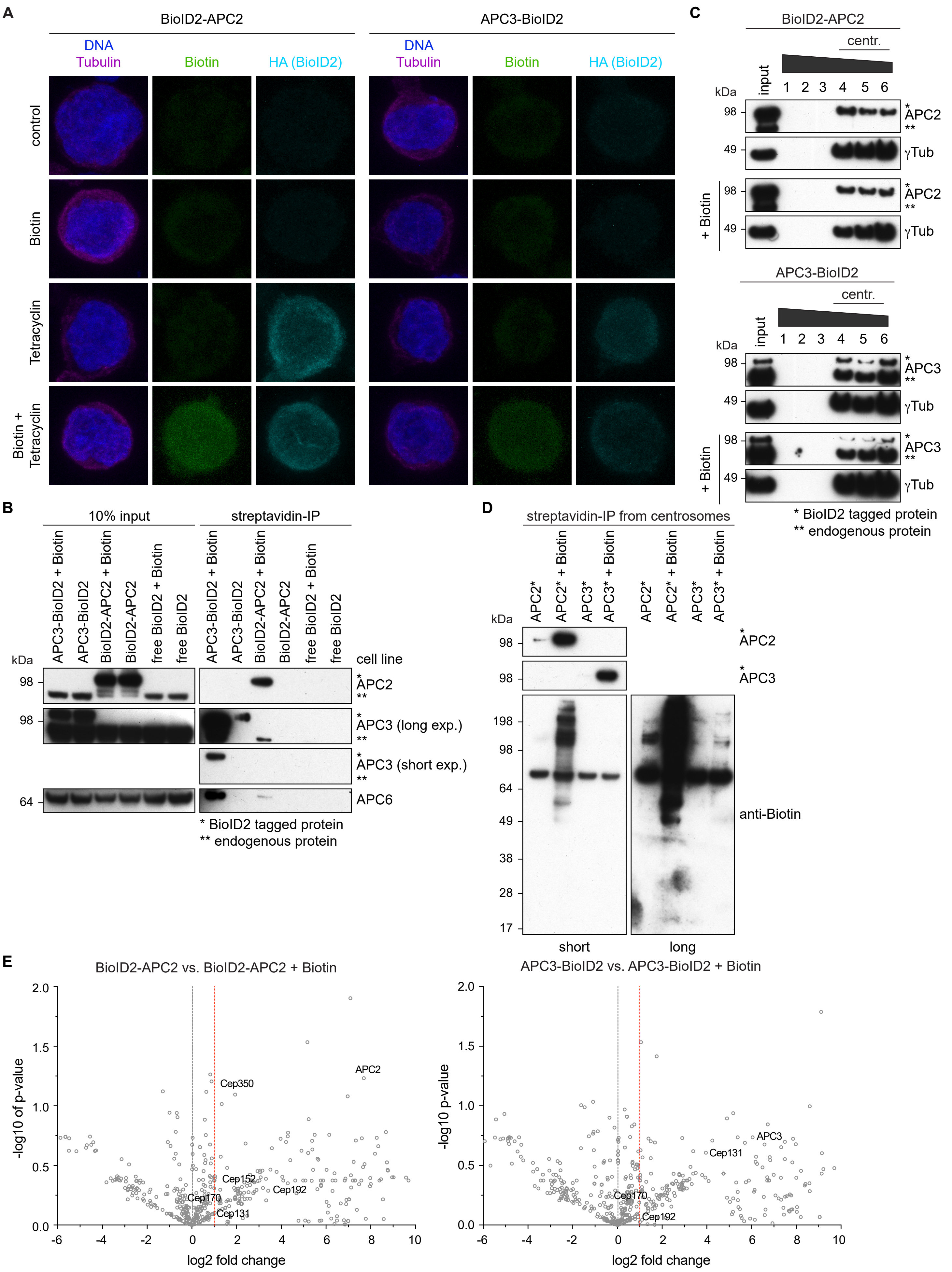

Fig. S3. The APC/C is in close proximity to several other centrosomal proteins. A , HEK293 FlpIn T-Rex cells with stable integration of the indicated constructs were treated with the indicated compounds. **B**, HEK293 FlpIn T-Rex cells were treated with tetracycline to induce expression of the stably integrated BioID2 constructs and with or without biotin for proximity labelling. Whole cell lysates were subjected to streptavidin pulldown to enrich for biotinylated proteins. The samples were immunoblotted against the indicated proteins. **C**, Cells were treated as in **B**, but additionally blocked in mitosis by nocodazole, and mitotic centrosomes were purified via a sucrose gradient. The first elution fractions were immunoblotted against γTubulin as a centrosome marker and the APC/C. **D**, The centrosome containing fractions from **c** were pooled and subsequently used for a streptavidin pulldown to enrich specifically for biotinylated centrosomal proteins. **E**, The streptavidin pulldown from **D** was subjected to mass-spectrometry and the data were evaluated by label free analysis. Proteins that were chosen for further analysis are indicated the graphs. See Supplementary Table 1 and 2 for the full mass-spec dataset.

Journal of Cell Science • Supplementary information

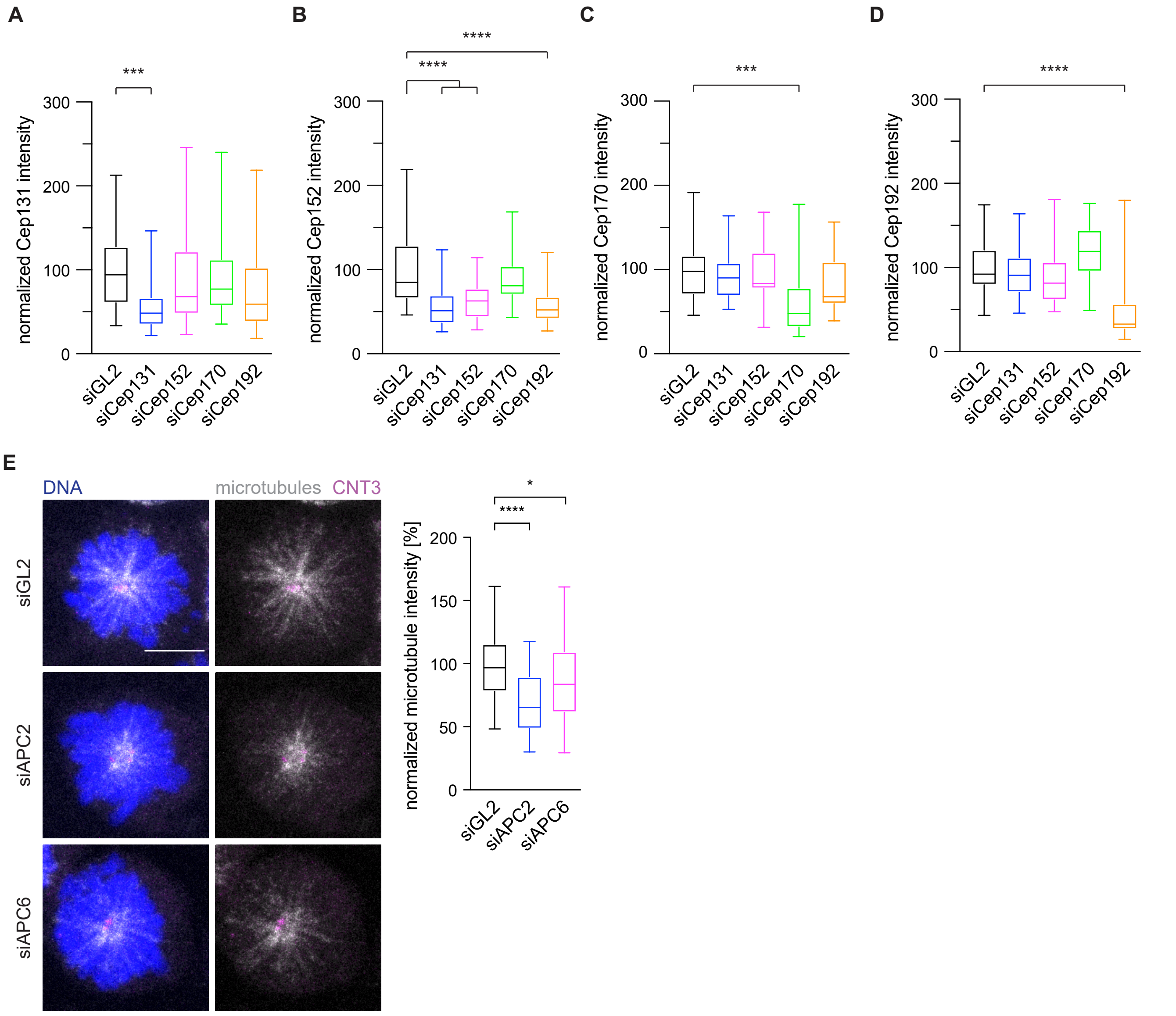

Fig. S4. Depletion and cross-staining of centrosomal proteins identified by BioID2 mass-spectrometry. A - D, HEK293 cells were depleted of the indicated centrosomal proteins using siRNA and arrested in mitosis by STLC. Cells were stained with the indicated antibodies and the centrosomal signal of each protein was quantified. The intensity was normalized against the siGL2 control. ****P < 0.0001 and ***P < 0.001 using a simple one-way ANOVA test with Dunnett's multiple comparison. **a**, N = 48 (siGL2), 57 (siCep131), 49 (siCep152), 37 (siCep170), 39 (siCep192). **B**, N = 51 (siGL2), 50 (siCep131), 40 (siCep152), 29 (siCep170), 50 (siCep192). **C**, N = 29 (siGL2), 33 (siCep131), 19 (siCep152), 29 (siCep170), 22 (siCep192). **D**, N = 51
(siGL2), 46 (siCep131), 44 (siCep152), 33 (siCep170), 38 (siCep192). **E**, HEK293 cell were treated with siRNA against APC2, APC6 or GL2 (control) and arrested in mitosis with STLC according to the time line shown in Figure **2A**. Cells were stained against the indicated proteins (left). Scale bars are 5 µm. The fluorescence intensity of tubulin in the whole cell was measured and normalized against the GL2 control (right). N = number of cells = 60 (siGL2), 60 (siAPC2), 62 (siAPC6). ***P = 0.0002, ****P < 0.0001 using a simple one-way ANOVA test with Dunnett's multiple comparison.

Journal of Cell Science • Supplementary information

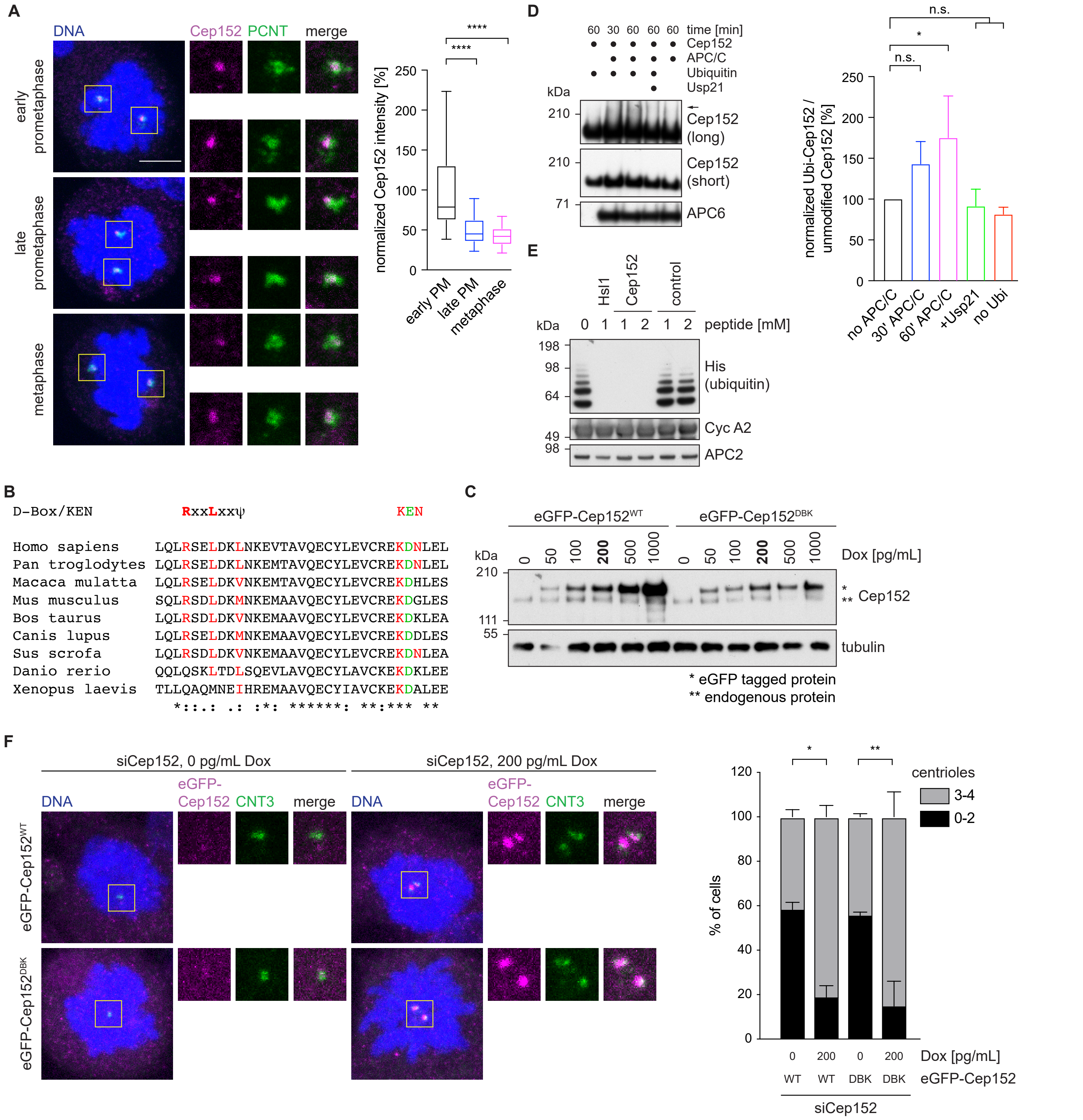

Fig. S5. Cep152 protein levels at the centrosome decrease during mitosis. A, HEK293 cells were treated according to the time line in **Figure 2A** and stained against the indicated proteins. The fluorescence intensity of Cep152 at the centrosome was measured and normalized against the intensity in prophase. Scale bars are 5 µm. N = 72 (prophase), 92 (pro-metaphase), 68 (metaphase). ****P < 0.0001 using a simple one-way ANOVA test with Dunnett's multiple comparison. **B**, Multiple sequence alignment of the Cep152 potential D box and KEN box. ψ = aliphatic aminoacids like GAVLMI. **C**, HEK293 Flp-In T-Rex cells carrying the indicated eGFP-Cep152 variants were treated with different concentrations of doxycycline for 24 h to induce the expression of the transgenes. Whole cell lysates were prepared and probed by immunoblot against the indicated proteins. Note that eGFP-Cep152 runs higher as the endogenous protein. For all other experiments 200 pg/mL doxycycline was used. **D**, eFGP-Cep152 was immunoprecipitated from HEK cells. The eluate was subjected to *in vitro* ubiquitination by incubation with purified APC/C in the presence or absence of ubiquitin or Usp21, where indicated (left). The intensity of the ubiquitinated bands was measured and normalized to the corresponding unmodified band (right). *P = 0.024 using a simple one-way ANOVA test with Dunnett's multiple comparison. N = number of immunoblots = three. Short exposure was 30 seconds, long exposure over 5 minutes. **E**, Cyclin A2 was ubiquitinated in vitro using purified APC/C in the presence of increasing concentrations of Hsl1 peptide, Cep152 peptide, or a scrambled control peptide respectively. **F**, HEK293 Flp-In T-Rex cells carrying the indicated eGFP-Cep152 variants were depleted of endogenous Cep152 by siRNA in the presence or absence of Doxycycline to induce the expression of the transgene according to the time line shown in **Figure 2B**. Cells were fixed with ice-cold methanol and stained with the indicated antibodies for immunofluorescence (left). The number of centrin-3 dots was counted and cells were classified as shown on the right. Depicted is the mean +/- s.d. *P < 0.05, **P < 0.01 using a two-way ANOVA test with Tukey's multiple comparison. N = number of experiments = two.

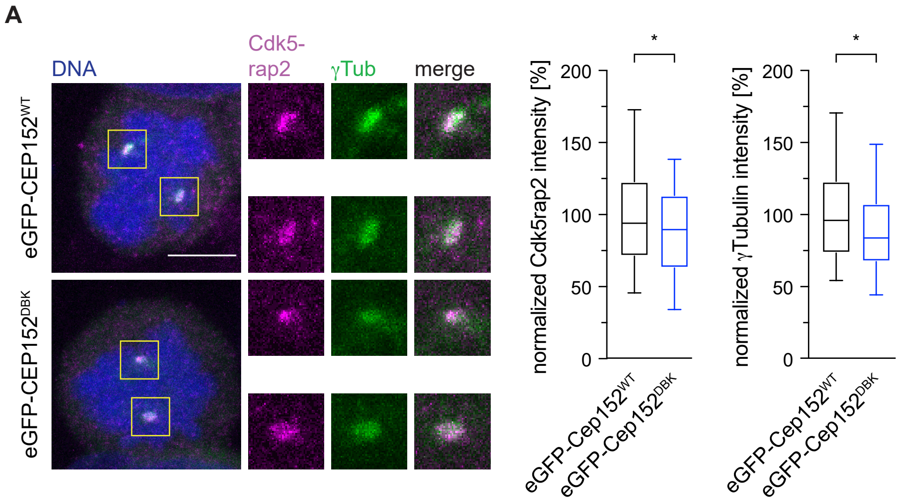

Fig. S6. Microtubule nucleation factors are reduced in the presence of stabilised Cep152. A, HEK293 FlpIn T-Rex cell lines expressing eGFP-Cep152 wildtype (WT) or D-box/KEN box (DBK) mutant proteins were treated as in **Figure 4A** and stained against the indicated proteins (left). The fluores-cence intensity of Cdk5rap2 (middle) and gamma-Tubulin (right) were measured at the centrosome and normalized against the eGFP-Cep152^{WT} cell line. Cdk5rap2: N = number of cells = 174 (WT), 179 (DBK), *P = 0.024 using a Mann-Whitney U-test. gamma-Tubulin: N = number of cells = 97 (WT), 98 (DBK), *P = 0.0341 using a Mann-Whitney U-test.

Table S1. APC2 and APC3 BioID2 Mass Spec data. Spectral counts and statistical analysis used to create Supplementary Figure 3E.

Click here to download Table S1

Table S2. APC2 and APC3 BioID2 Mass Spec data. Summary of all two-fold or higher enriched proteins. Selected centrosomal proteins for further analysis are marked in red and bait proteins are marked in green.

Click here to download Table S2

Journal of Cell Science • Supplementary information

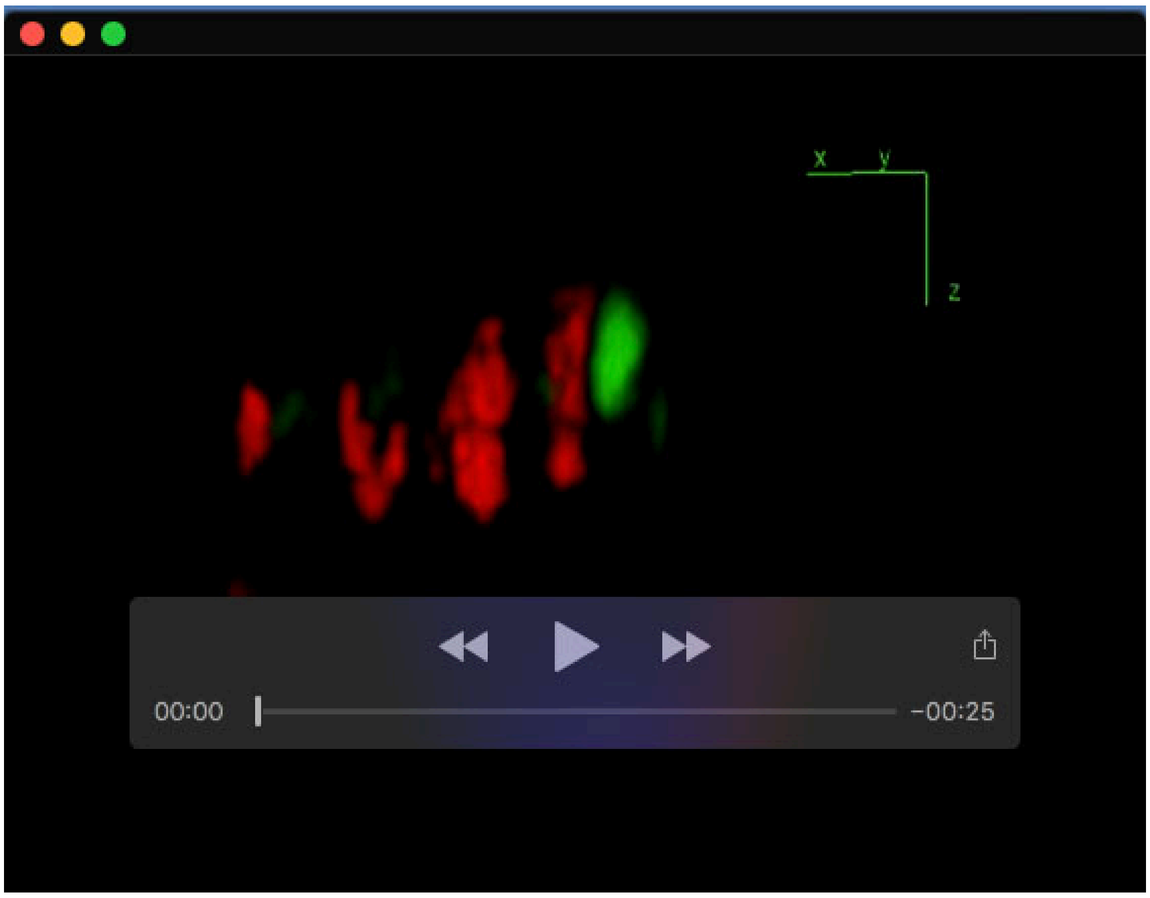

Movie 1. STORM imaging of centrosomes showing APC2 and Cent3 localization, related to Supplementary Figure S2E (top).

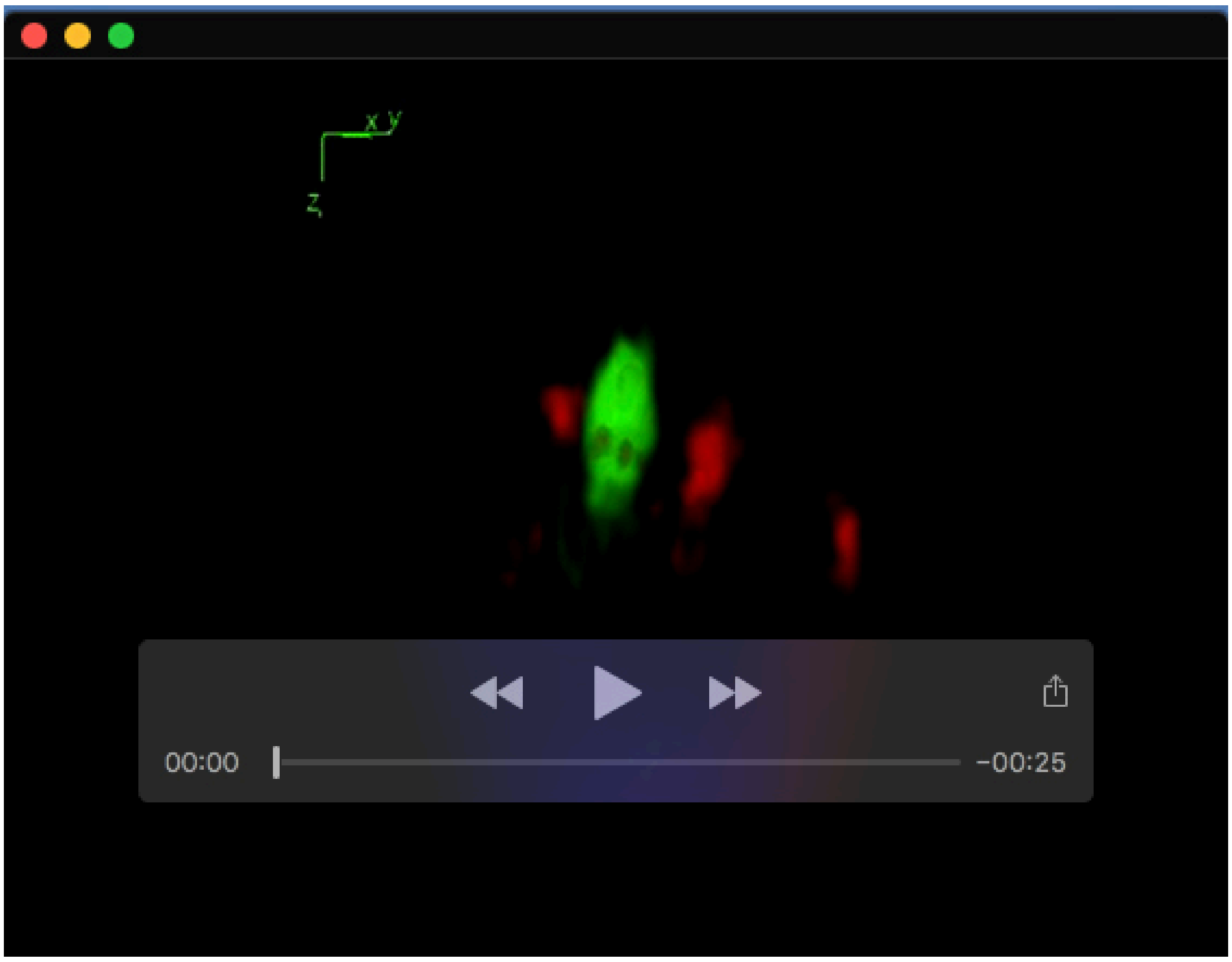

Movie 2. STORM imaging of centrosomes showing APC2 and Cent3 localization, related to Supplementary Figure S2E (bottom).

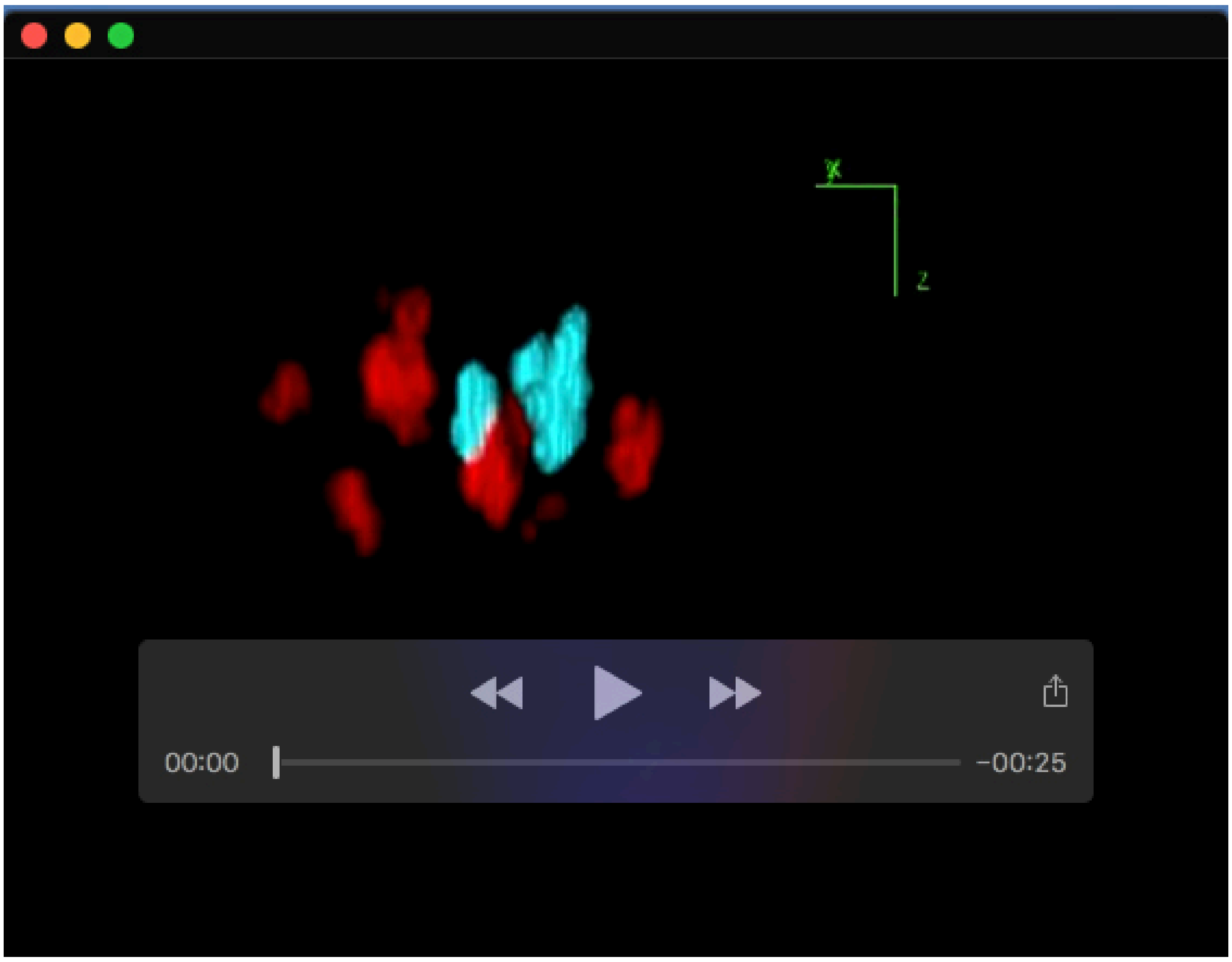

Movie 3. STORM imaging of centrosomes showing APC2 and C-Nap1 localization, related to Supplementary Figure S2F (top).

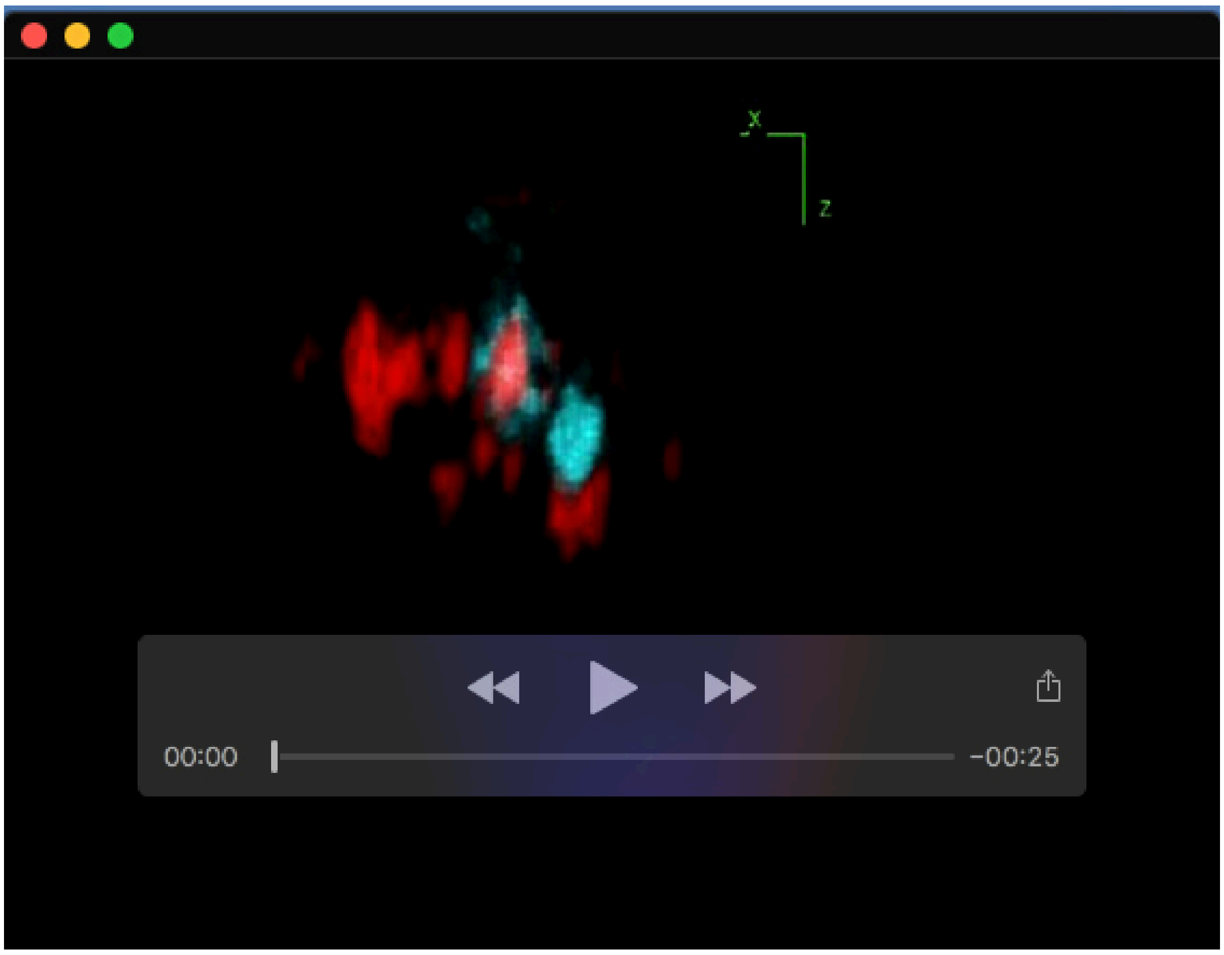

Movie 4. STORM imaging of centrosomes showing APC2 and C-Nap1 localization, related to Supplementary Figure S2F (bottom).

Journal of Cell Science • Supplementary information

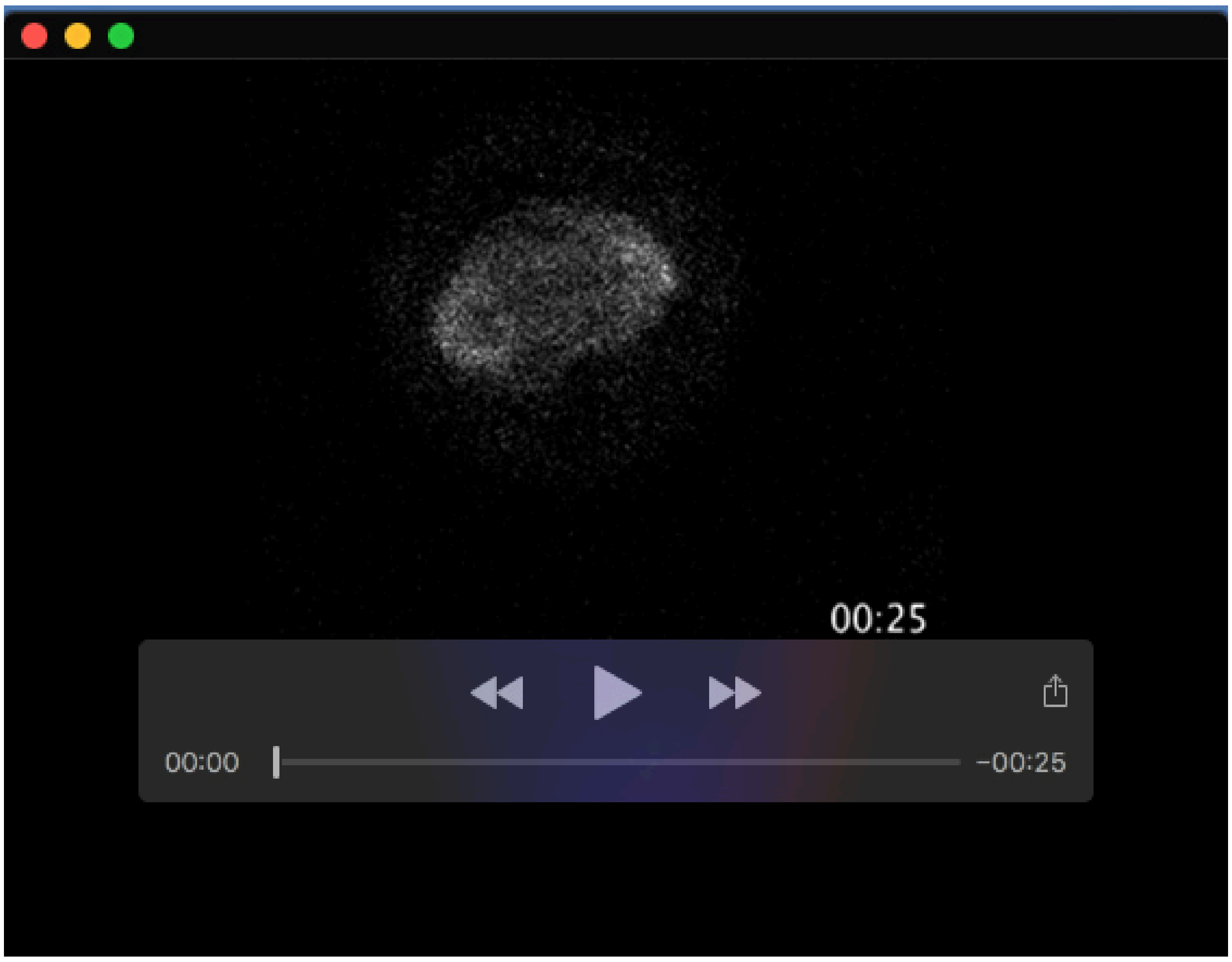

Movie 5. Live cell imaging of HEK293 Flp-In TRex cells expressing eGFP-Cep152^{WT}, microtubules are labelled with SiR-tubulin, related to Figure 6A (top).

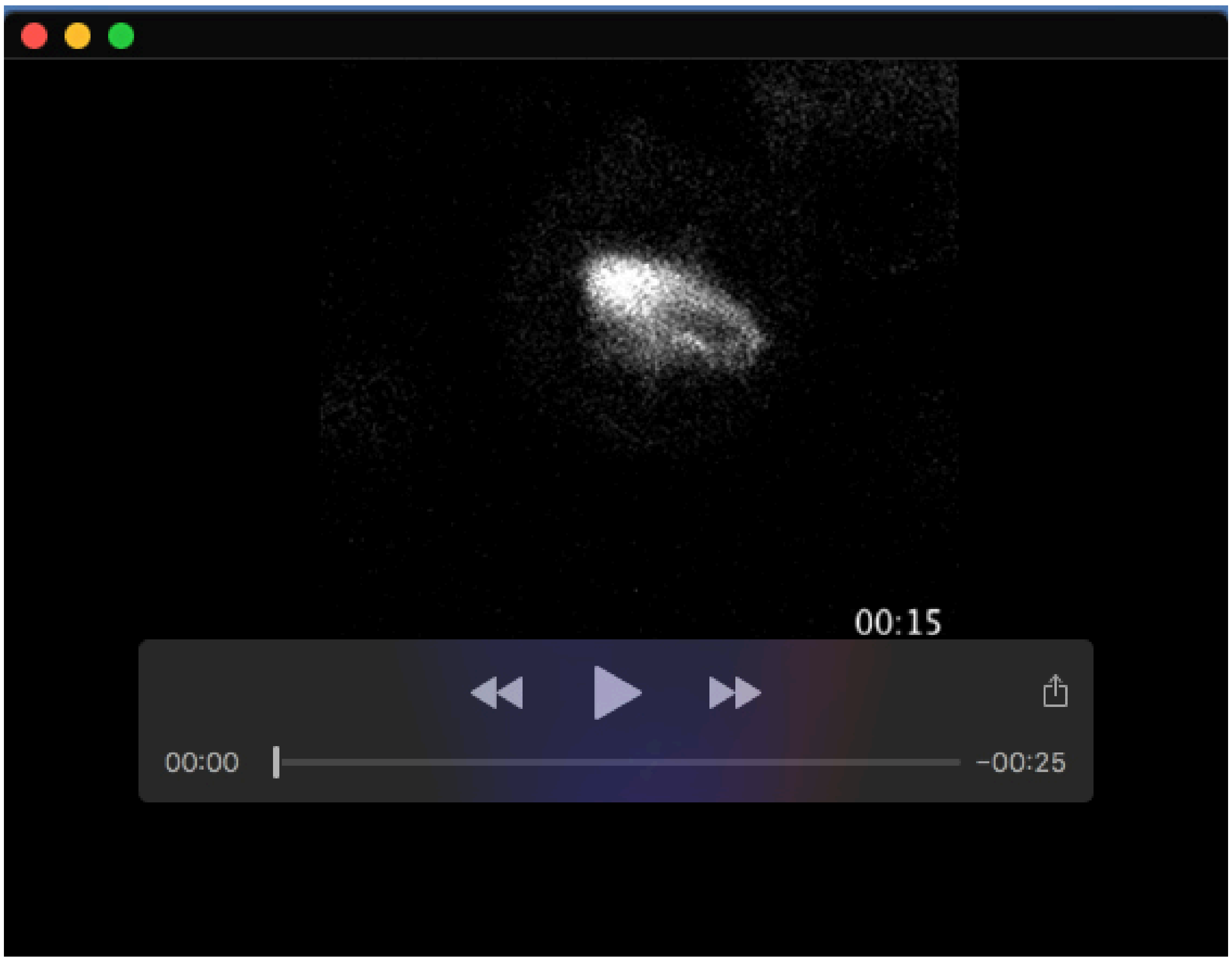

Movie 6. Live cell imaging of HEK293 Flp-In TRex cells expressing eGFP-Cep152^{DBK}, microtubules are labelled with SiR-tubulin, related to Figure 6A (bottom).

Journal of Cell Science • Supplementary information

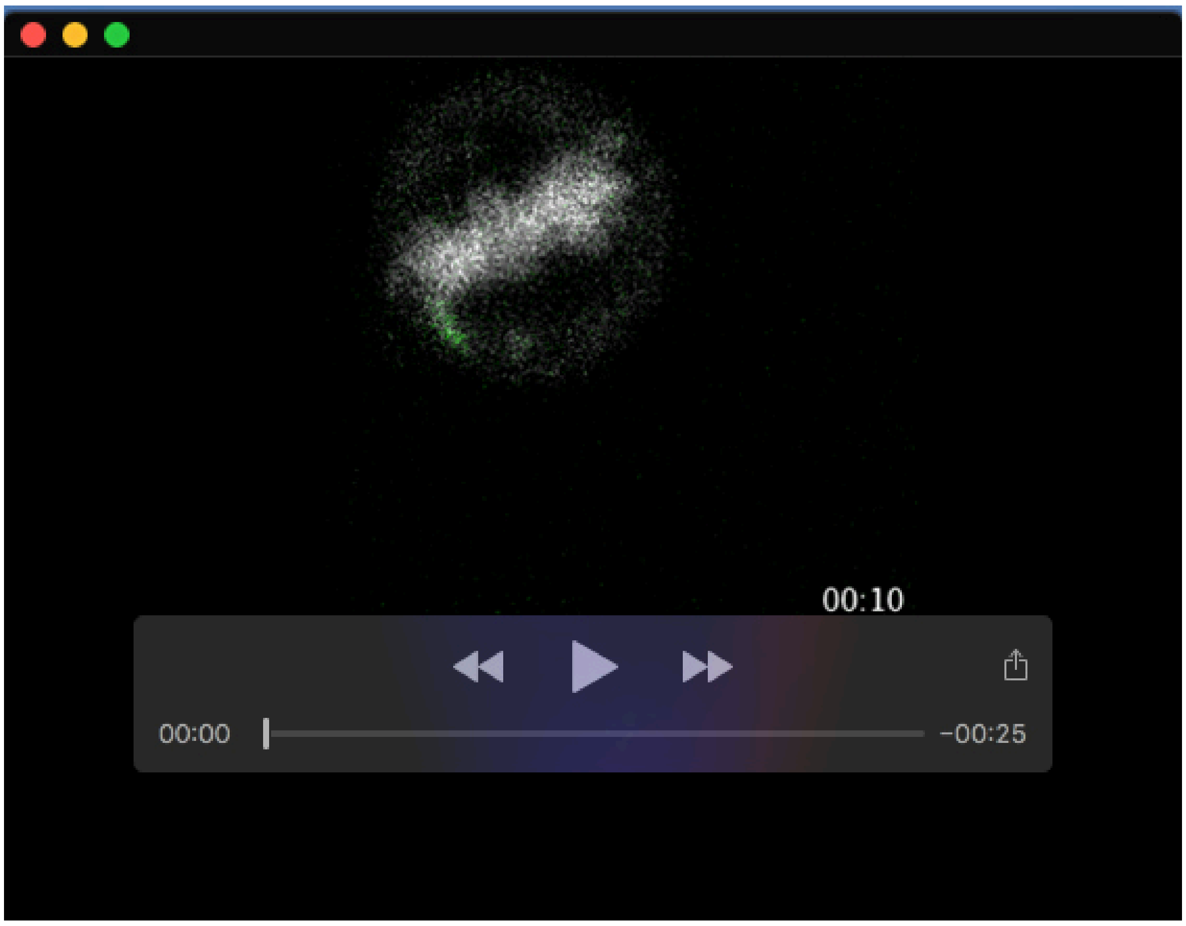

Movie 7. Live cell imaging of HEK293 Flp-In TRex cells expressing eGFP-Cep152^{WT}, DNA is labelled with SiR-DNA, related to Figure 6B (top).

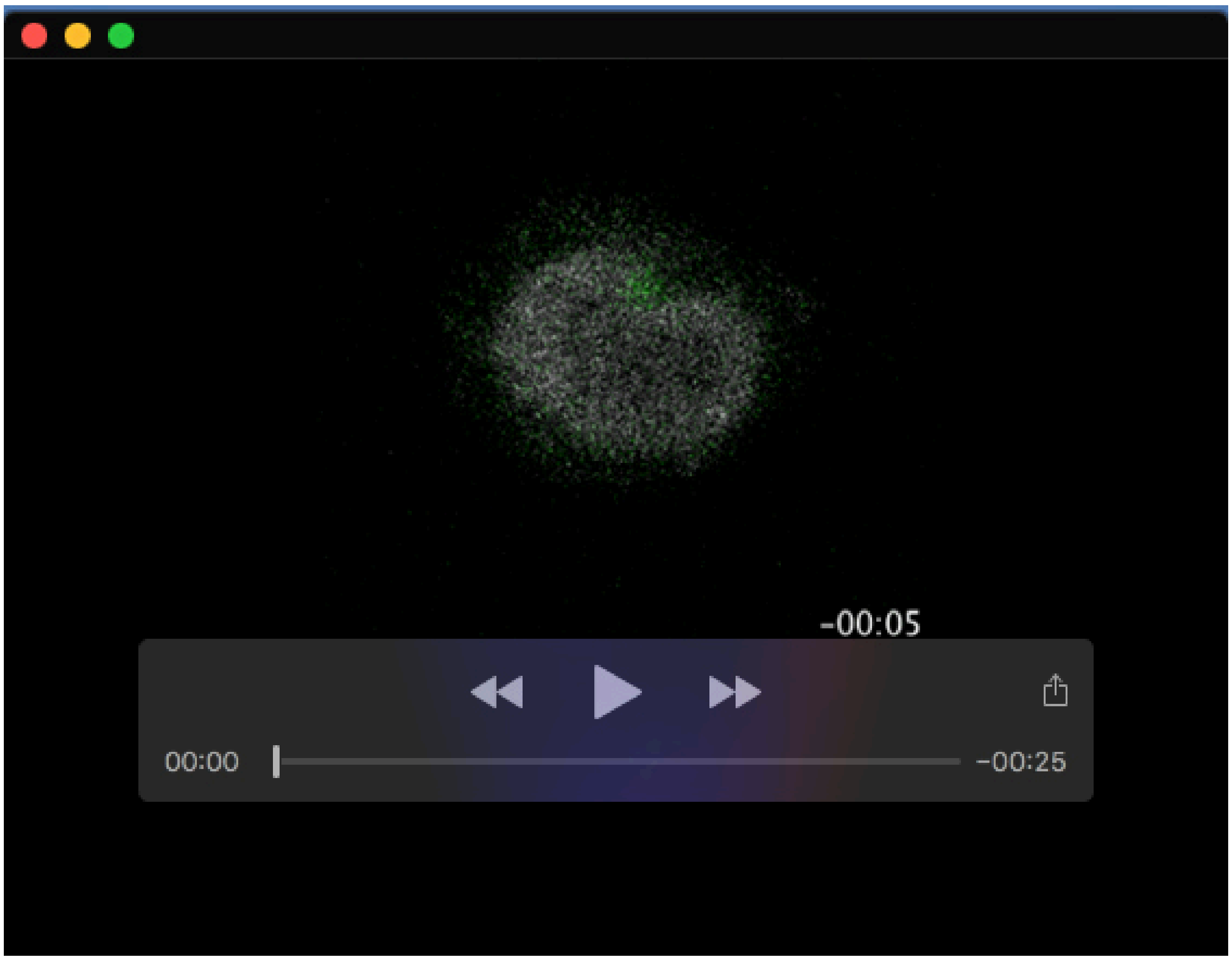

Movie 8. Live cell imaging of HEK293 Flp-In TRex cells expressing eGFP-Cep152^{DBK}, DNA is labelled with SiR-DNA, related to Figure 6B (bottom).

Journal of Cell Science • Supplementary information
