# Supplementary material for: The APC/C targets the Cep152–Cep63 complex at the centrosome to regulate mitotic spindle assembly
Source: J Cell Sci. 2022 Jan 18;135(2):jcs259273. doi: 10.1242/jcs.259273 (PMC8917351; doi:10.1242/jcs.259273)
Supplement: Reviewer comments [file joces-135-259273_review_history.pdf]

The APC/C targets the Cep152-Cep63 complex at the centrosome to regulate mitotic spindle assembly

Thomas Tischer, Jing Yang and David Barford
DOI: 10.1242/jcs.259273

Editor: David Glover

Review timeline
Original submission: 13 August 2021
Editorial decision: 6 October 2021
First revision received: 20 October 2021
Accepted: 25 November 2021

Original submission

First decision letter

MS ID#: JOCES/2021/259273

MS TITLE: The APC/C removes the Cep152-Cep63 complex from the centrosome to regulate mitotic spindle assembly

AUTHORS: Thomas Tischer, Jing Yang, and David Barford
ARTICLE TYPE: Research Article

We have now reached a decision on the above manuscript.

To see the reviewers' reports and a copy of this decision letter, please go to: https://submit-jcs.biologists.org and click on the 'Manuscripts with Decisions' queue in the Author Area. (Corresponding author only has access to reviews.)

As you will see, the reviewers raise a number of substantial criticisms that prevent me from accepting the paper at this stage. They suggest, however, that a revised version might prove acceptable, if you can address their concerns. If you think that you can deal satisfactorily with the criticisms on revision, I would be pleased to see a revised manuscript. We would then return it to the reviewers.

We are aware that you may be experiencing disruption to the normal running of your lab that makes experimental revisions challenging. If it would be helpful, we encourage you to contact us to discuss your revision in greater detail. Please send us a point-by-point response indicating where you are able to address concerns raised (either experimentally or by changes to the text) and where you will not be able to do so within the normal timeframe of a revision. We will then provide further guidance. Please also note that we are happy to extend revision timeframes as necessary.

Please ensure that you clearly highlight all changes made in the revised manuscript. Please avoid using 'Tracked changes' in Word files as these are lost in PDF conversion.

I should be grateful if you would also provide a point-by-point response detailing how you have dealt with the points raised by the reviewers in the 'Response to Reviewers' box. Please attend to all of the reviewers' comments. If you do not agree with any of their criticisms or suggestions please explain clearly why this is so.

© 2021. Published by The Company of Biologists under the terms of the Creative Commons Attribution License (https://creativecommons.org/licenses/by/4.0/).

Reviewer 1

Advance summary and potential significance to field

Tischer et al. studied the role of the cell cycle ubiquitin ligase APC/C in the regulation of centrosomes and spindle assembly in cultured human cells. It was shown that some of APC/C subunits are localised near the centrosome accumulating during mitosis. This localisation depends on a conserved centrosome component, CEP152. CEP152 and APC/C interacted with each other; whether the interaction is direct or indirect is not determined. The authors also provide evidence that CEP152 is also a substrate of APC/C-dependent ubiquitination. The mutant forms of CEP152, which had its putative APC/C degrons mutated accumulates at mitotic centrosomes and causes a delay in mitotic progression accompanying chromosome misalignment and lagging chromosomes. Furthermore, in this condition, the interaction between CEP57 and PCNT is reduced, leading to a model where the ubiquitination of CEP152 by APC/C allows CEP57 to dissociate from the CEP152-CEP63 inhibitory complex and bind PCNT to form the spindle.

Based on these observations, the authors propose that APC/C regulates spindle assembly by promoting the microtubule nucleation activity of the centrosome through the regulation of CEP152 levels at the centrosomes.

This study contains novel and important observations about centrosomes and cell cycle control mechanisms. In particular, the study provides new molecular insight into the mechanism that controls the conversion of the centrosome from the interphase form to the mitotic form through ubiquitin-dependent proteolysis as well as the spatial regulation of the functions of APC/C. Their proposed model for the role of CEP152 degradation in the conversion of the centrosome is straight-forward and quite attractive, which is well supported by their experimental data.

Comments for the author

For the reasons mentioned above I fully support this paper for publication in JSC after revisions suggested below.
- “We identified BubR1 in centrosomal fractions, but did not observe co-elution of the other SAC proteins Mad2 or Bub3 (Figure 1e)” ↳ Should be 1d.
- In Figure 2, although the quantification of APC2 signal intensity at the centrosome shows a clear decrease with the progression into mitosis, the IF images are not so clear to me. In metaphase, APC2 signal looks probably less extended than in early and late PM (less background) but the signal intensity seems similar, maybe higher, in metaphase in this figure (particularly in the top image of the metaphase panel). Maybe better to choose more representative images.
- Fig2C: Cep350 ↳ APC2 signal is much lower than in the control condition but the text: “except for Cep350 where APC/C intensity at the centrosome remained unchanged”. Maybe better to choose more representative image then.
- Figure 3a legend: “HEK293 cells were treated…”
- Figure 3c: “GFP152WT ubiquitination is suppressed by the small APC/C inhibitors TAME and APCin, demonstrating that Cep152 ubiquitination is APC/C-dependent.” In fig 3c, we can see that Cep152WT ubiquitination decreases with the inhibitors, but it is not completely suppressed. APC/C inhibition is not complete, or other E3 ligases target Cep152 additionally to APC/C? Can the authors explain?
- Fig 4d legend : “[…] mutant proteins were treated according to the schedule in c and fixed to assess microtubule stability.” Idem in Fig 4e
- Fig 6a legend: “Microtubule intensity was measured in two separate…”
- Fig 6f: Is it possible to add PCNT and Cep63 to this diagram? I think the difference between normal mitosis and stabilized Cep152 conditions would be more obvious this way.
- In the description about 2D and 3D-dSTORM in the Methods section: “The secondary antibodies used were coupled to Alexafluor-568 or Alexafluor-647”
- References: I didn’t find the Ref for Zhang et al, 2016 in the text.

© 2021. Published by The Company of Biologists under the terms of the Creative Commons Attribution License (https://creativecommons.org/licenses/by/4.0/).

Reviewer 2

Advance summary and potential significance to field

The authors demonstrated that APC/C localizes to the centrosome during mitosis. The most notable discovery however is that they show evidence that Cep152 is responsible for recruiting APC/C to the centrosome in mitosis and is itself a substrate of APC/C.
A weakness authors are also aware of is that they cannot show a direct interaction between Cep152 and APC/C. However, this is blunted to some extent with the series of experiments that altogether point to the Cep152 beeing and APC/C substrate: TAME/APCin inhibitors abolish Cep152 ubiquitylation, KEN-box/D-box mutant version of Cep152 is not ubiquitylated; in vitro ubiquitylation assay of GFP-Cep152 purified from cells show shift upon APC/C addition; Ub-competition assay with Cep152 DBK based peptide abolished cyclin A2 ubiquitylation.
A notable observation is that stabilization of Cep152 at the centrosomes during mitosis phenocopies APC/C mutations by showing reduced spindle microtubule intensity and PCNT intensity. Their follow-up experiments showed a delay in microtubule nucleation and establishing a bipolar spindle. Additional experiments demonstrated that Cep152 acts as an inhibitor of microtubule formation by forming a complex with Cep63 to inhibit Cep57. Preventing Cep152 degradation by eliminating its KEN and D-box will lead to the observation of mitotic errors. Taken together, a series of properly performed experiments support the authors statements. It is also noteworthy that the authors do not overinterpret their data, for example they are mindful that Cep152 doesn't behave like a very strong APC/C substrate – which can be explained by the fact that it is ubiquitylated in a well-defined spatiotemporal context (at the centrosome in mitosis). I think this paper is a very good addition to the knowledge of APC/C function at the centrosome and I suggest being accepted for publication is JCS as it is.

Comments for the author

The authors demonstrated that APC/C localizes to the centrosome during mitosis. The most notable discovery however is that they show evidence that Cep152 is responsible for recruiting APC/C to the centrosome in mitosis and is itself a substrate of APC/C.
A weakness authors are also aware of is that they cannot show a direct interaction between Cep152 and APC/C. However, this is blunted to some extent with the series of experiments that altogether point to the Cep152 beeing and APC/C substrate: TAME/APCin inhibitors abolish Cep152 ubiquitylation, KEN-box/D-box mutant version of Cep152 is not ubiquitylated; in vitro ubiquitylation assay of GFP-Cep152 purified from cells show shift upon APC/C addition; Ub-competition assay with Cep152 DBK based peptide abolished cyclin A2 ubiquitylation.
A notable observation is that stabilization of Cep152 at the centrosomes during mitosis phenocopies APC/C mutations by showing reduced spindle microtubule intensity and PCNT intensity. Their follow-up experiments showed a delay in microtubule nucleation and establishing a bipolar spindle. Additional experiments demonstrated that Cep152 acts as an inhibitor of microtubule formation by forming a complex with Cep63 to inhibit Cep57. Preventing Cep152 degradation by eliminating its KEN and D-box will lead to the observation of mitotic errors. Taken together, a series of properly performed experiments support the authors statements. It is also noteworthy that the authors do not overinterpret their data, for example they are mindful that Cep152 doesn't behave like a very strong APC/C substrate – which can be explained by the fact that it is ubiquitylated in a well-defined spatiotemporal context (at the centrosome in mitosis). I think this paper is a very good addition to the knowledge of APC/C function at the centrosome and I suggest being accepted for publication is JCS as it is.

Reviewer 3

Advance summary and potential significance to field

In the current manuscript Tischer, Yang and Barford propose a new role for the Anaphase-Promoting-Complex/Cyclosome (APC/C) in mitotic spindle formation. Having verified a previously observed localisation of APC/C at the spindle poles (or in the centrososmal PCM), they go on to functionally dissect the mechanisms of its recruitment there, as well as its function in that location. Using proximity labelling assay, coupled with mass-spectrometry, the authors identify several centrosomal partners of the APC/C, amongst which Cep152. They show that APC/C is

© 2021. Published by The Company of Biologists under the terms of the Creative Commons Attribution License (https://creativecommons.org/licenses/by/4.0/).

dependent for its PCM localisation on Cep152, but also that Cep152 is an ubiquitination substrate of APC/C. Furthermore, Tischer Yang and Barford create a non-ubiquitilatable DEAD-box and KEN-box (DBK) mutant version of Cep152, which does not show an APC/C-dependent reduction on mitotic centrosomes like its WT counterpart. Using the DBK-mutant version, they then demonstrate that a failure to target Cep152 for degradation/release from the centrosome results in reduced PCM, less robust microtubule spindle and, eventually in mitotic errors. The authors then propose that these phenotypes are a result of an inhibitory complex consisting of Cep152-Cep63-Cep57. In normal (WT) situation, this complex would be destroyed/disassembled upon Cep152 targeting by the APC/C, releasing Cep57 from this inhibition. Cep57 would then be free to recruit the PCM factor PCNT and enhance PCM recruitment and MT-nucleation.

This work is very interesting and important as it proposes new mitotic roles for both the APC/C and Cep152. The manuscript is very well written, the experiments are thorough and for the most part the results justify the conclusions drawn by the authors. I would recommend it for publication in JCS, provided the authors respond to all of my comments listed below.

Comments for the author

1) Starting with the title, I would suggest that the authors soften/change their language at places. The word “removes” in the title for example, used without any context or explanation, implies a complete removal of Cep152, which is simply not the case. In all experiments that address this, there is a significant portion of Cep152 left on the centrososme, and in some cases the reduction is less than 50%... A more appropriate term would be “targets” or using others like “reduction”, “attenuation”, etc.
2) Page 3: “APC/C subunits are rarely present in isolation, which means the detection of one or two subunits of the complex argues for the presence of the whole E3 ligase”. The authors do not need to “argue”, they can co-stain APC/C-subunits in pair-wise fashion and demonsrate extensive colocalisation between them at PCM/centrosomes. Similarly, and especially as they base all further IF of APC/C on a single APC-2 antibody, they should try to verify at least some of their key results with another anti-APC-2 Ab or, failing that, with an Ab against other APC/C subunit.
3) Notably missing from this work is a co-localisation between APC/C and Cep152. Considering these are the main molecules studied here (and their physical and functional interplay), this is a must. One would expect at least a partial co-localisation between them, considering that they are suggested to interact and one to be a substrate of the other.
4) The most downstream and most-important PCM component (at least in terms of MT-nucleation) is gamma-Tubulin (complexes). So I think the authors need to use anti-gamma-Tubulin Ab in all the experiments where they study/quantify the effects on PCM-recruitment. Not instead of PCNT, but in addition to.
5) In several places where immunoblots are used for drawing conclusions on comparative strength of interactions or levels of ubiquitination, there is no quantification provided. This should be corrected and normalisation by inputs should be done where relevant. The figures in question are Fig. 3C; Fig. 5C; Suppl.Fig. 5D. Please, check for others I may have missed.

Minor points:
1) Page 5: In several places the descriptive “APC/C molecules” is used. If I understand correctly, the authors mean instead the whole multimolecular APC/C scaffold/complex, which cannot be referred to as a “molecule”. Please, correct it.
2) Page 7: “To generate a small-scale interaction network…”. Use instead “dependency/hierarchy network” as this is what you are testing for, not interactions.
3) Page 8: “A concentration of 200 ng/mL tetracycline…”. In all the figures pg/ml is used. Which one is correct?
4) Page 10: “…less microtubules…” Correct to “…fewer microtubules…”

© 2021. Published by The Company of Biologists under the terms of the Creative Commons Attribution License (https://creativecommons.org/licenses/by/4.0/).

First revision

Author response to reviewers' comments

Reviewer 1
Advance Summary and Potential Significance to Field:

Tischer et al. studied the role of the cell cycle ubiquitin ligase APC/C in the regulation of centrosomes and spindle assembly in cultured human cells. It was shown that some of APC/C subunits are localised near the centrosome, accumulating during mitosis. This localisation depends on a conserved centrosome component, CEP152. CEP152 and APC/C interacted with each other; whether the interaction is direct or indirect is not determined. The authors also provide evidence that CEP152 is also a substrate of APC/C-dependent ubiquitination. The mutant forms of CEP152, which had its putative APC/C degrons mutated, accumulates at mitotic centrosomes and causes a delay in mitotic progression, accompanying chromosome misalignment and lagging chromosomes. Furthermore, in this condition, the interaction between CEP57 and PCNT is reduced, leading to a model where the ubiquitination of CEP152 by APC/C allows CEP57 to dissociate from the CEP152-CEP63 inhibitory complex and bind PCNT to form the spindle. Based on these observations, the authors propose that APC/C regulates spindle assembly by promoting the microtubule nucleation activity of the centrosome through the regulation of CEP152 levels at the centrosomes. This study contains novel and important observations about centrosomes and cell cycle control mechanisms. In particular, the study provides new molecular insight into the mechanism that controls the conversion of the centrosome from the interphase form to the mitotic form through ubiquitin-dependent proteolysis, as well as the spatial regulation of the functions of APC/C. Their proposed model for the role of CEP152 degradation in the conversion of the centrosome is straight-forward and quite attractive, which is well supported by their experimental data.

Reviewer 1
Comments for the Author:

For the reasons mentioned above I fully support this paper for publication in JSC after revisions suggested below.
- “We identified BubR1 in centrosomal fractions, but did not observe co-elution of the other SAC proteins Mad2 or Bub3 (Figure 1e)” ⤹ Should be 1d.
We thank the reviewer for spotting this mistake. Now corrected.

- In Figure 2, although the quantification of APC2 signal intensity at the centrosome shows a clear decrease with the progression into mitosis, the IF images are not so clear to me. In metaphase, APC2 signal looks probably less extended than in early and late PM (less background) but the signal intensity seems similar, maybe higher, in metaphase in this figure (particularly in the top image of the metaphase panel). Maybe better to choose more representative images.
We agree that the images did not fully represent the quantification. We therefore replaced them with better fitting cells.

- Fig2C: Cep350 ⤹ APC2 signal is much lower than in the control condition but the text: “except for Cep350 where APC/C intensity at the centrosome remained unchanged”. Maybe better to choose more representative image then.
We replaced the image in Figure 2C representing the Cep350 depletion phenotype with another cell that more closely reflects the quantification.

- Figure 3a legend: “HEK293 cells were treated…”
We thank the reviewer for spotting this typo. Now corrected.

- Figure 3c: “GFP152WT ubiquitination is suppressed by the small APC/C inhibitors TAME and APCin, demonstrating that Cep152 ubiquitination is APC/C- dependent.” In fig 3c, we can see that Cep152WT ubiquitination decreases with the inhibitors, but it is not completely suppressed. APC/C inhibition is not complete, or other E3 ligases target Cep152 additionally to APC/C? Can the authors explain?

© 2021. Published by The Company of Biologists under the terms of the Creative Commons Attribution License (https://creativecommons.org/licenses/by/4.0/).

We agree with the reviewer that eGFP-Cep152^{WT} ubiquitination is not completely suppressed by the addition of APC/C inhibitors. Both suggestions from the reviewer could be correct, however we favour the explanation that APC/C is not inhibited to 100%. Our reasoning is that the combination of eGFP-Cep152^{DBK}, which is already resistant to APC/C mediated ubiquitination, with additional APC/C inhibitors leads to a near complete inhibition of ubiquitination. Since we cannot formally exclude that other ubiquitin ligases could also target Cep152, we added a sentence on page 9 to explain the observed behaviour.

- Fig 4d legend : "[…] mutant proteins were treated according to the schedule in c and fixed to assess microtubule stability." Idem in Fig 4e
We apologize for this typo. Now corrected.

- Fig 6a legend: "Microtubule intensity was measured in two separate…"
We thank the reviewer for noticing this typo. Now corrected.

- Fig 6f: Is it possible to add PCNT and Cep63 to this diagram? I think the difference between normal mitosis and stabilized Cep152 conditions would be more obvious this way.
We added Cep63 to the model and indicated that the blue background indicates PCNT (together with other PCM proteins).

- In the description about 2D and 3D-dSTORM in the Methods section: "The secondary antibodies used were coupled to Alexafluor-568 or Alexafluor-647"
To clarify this, we changed the sentence to "The secondary antibodies were pre-coupled to Alexafluor-568 or Alexafluor-647 (Thermofisher, 1:500)…"

- References: I didn't find the Ref for Zhang et al, 2016 in the text.
The reference to Zhang et al, 2016 is in the Material and Methods section on page 18.

Reviewer 2 Advance Summary and Potential Significance to Field:

The authors demonstrated that APC/C localizes to the centrosome during mitosis. The most notable discovery however is that they show evidence that Cep152 is responsible for recruiting APC/C to the centrosome in mitosis and is itself a substrate of APC/C.

A weakness authors are also aware of is that they cannot show a direct interaction between Cep152 and APC/C. However, this is blunted to some extent with the series of experiments that altogether point to the Cep152 beeing and APC/C substrate: TAME/APCin inhibitors abolish Cep152 ubiquitylation, KEN-box/D-box mutant version of Cep152 is not ubiquitylated; in vitro ubiquitylation assay of GFP-Cep152 purified from cells show shift upon APC/C addition; Ub-competition assay with Cep152 DBK based peptide abolished cyclin A2 ubiquitylation.

A notable observation is that stabilization of Cep152 at the centrosomes during mitosis phenocopies APC/C mutations by showing reduced spindle microtubule intensity and PCNT intensity. Their follow-up experiments showed a delay in microtubule nucleation and establishing a bipolar spindle. Additional experiments demonstrated that Cep152 acts as an inhibitor of microtubule formation by forming a complex with Cep63 to inhibit Cep57. Preventing Cep152 degradation by eliminating its KEN and D-box will lead to the observation of mitotic errors. Taken together, a series of properly performed experiments support the authors statements. It is also noteworthy that the authors do not overinterpret their data, for example they are mindful that Cep152 doesn't behave like a very strong APC/C substrate – which can be explained by the fact that it is ubiquitylated in a well-defined spatiotemporal context (at the centrosome in mitosis). I think this paper is a very good addition to the knowledge of APC/C function at the centrosome and I suggest being accepted for publication is JCS as it is.

We thank the reviewer for their positive evaluation of our manuscript, and appreciate their time and effort assessing it.

© 2021. Published by The Company of Biologists under the terms of the Creative Commons Attribution License (https://creativecommons.org/licenses/by/4.0/).

Reviewer 3 Advance Summary and Potential Significance to Field:

In the current manuscript Tischer, Yang and Barford propose a new role for the Anaphase-Promoting-Complex/Cyclosome (APC/C) in mitotic spindle formation. Having verified a previously observed localisation of APC/C at the spindle poles (or in the centrososmal PCM), they go on to functionally dissect the mechanisms of its recruitment there, as well as its function in that location. Using proximity labelling assay, coupled with mass-spectrometry, the authors identify several centrosomal partners of the APC/C, amongst which Cep152. They show that APC/C is dependent for its PCM localisation on Cep152, but also that Cep152 is an ubiquitination substrate of APC/C. Furthermore, Tischer Yang and Barford create a non-ubiquitilatable DEAD-box and KEN-box (DBK) mutant version of Cep152, which does not show an APC/C-dependent reduction on mitotic centrosomes like its WT counterpart. Using the DBK-mutant version, they then demonstrate that a failure to target Cep152 for degradation/release from the centrosome results in reduced PCM, less robust microtubule spindle and, eventually in mitotic errors. The authors then propose that these phenotypes are a result of an inhibitory complex consisting of Cep152-Cep63-Cep57. In normal (WT) situation, this complex would be destroyed/disassembled upon Cep152 targeting by the APC/C, releasing Cep57 from this inhibition. Cep57 would then be free to recruit the PCM factor PCNT and enhance PCM recruitment and MT-nucleation.

This work is very interesting and important as it proposes new mitotic roles for both the APC/C and Cep152. The manuscript is very well written, the experiments are thorough and for the most part the results justify the conclusions drawn by the authors. I would recommend it for publication in JCS, provided the authors respond to all of my comments listed below.

Reviewer 3 Comments for the Author:
1) Starting with the title, I would suggest that the authors soften/change their language at places. The word “removes” in the title for example, used without any context or explanation, implies a complete removal of Cep152, which is simply not the case. In all experiments that address this, there is a significant portion of Cep152 left on the centrososme, and in some cases the reduction is less than 50%... A more appropriate term would be “targets” or using others like “reduction”, “attenuation”, etc.

We agree with this evaluation. Cep152 is targeted by the APC/C, that results in reduced levels of Cep152, and Cep63, rather than their complete removal. We have therefore changed the title of our manuscript to: ‘The APC/C targets the Cep152-63 complex at the centrosome to regulate mitotic spindle assembly’. Additionally, we changed the text as suggested to clarify that levels of Cep152 and Cep63 are reduced.

2) Page 3: “APC/C subunits are rarely present in isolation, which means the detection of one or two subunits of the complex argues for the presence of the whole E3 ligase”. The authors do not need to “argue”, they can co-stain APC/C-subunits in pair-wise fashion and demonstrate extensive colocalisation between them at PCM/centrosomes. Similarly, and especially as they base all further IF of APC/C on a single APC-2 antibody, they should try to verify at least some of their key results with another anti-APC-2 Ab or, failing that, with an Ab against other APC/C subunit.

There are three key observations in our manuscript: (1) The APC/C localizes to centrosomes directly and not only to the area around spindle poles. (2) Cep152 is a substrate of the APC/C and (3) prolonged Cep152 presence at the centrosome delays microtubule nucleation in mitosis. Only the first observation relies on immunofluorescence staining using the APC2 antibody. We specifically verified this antibody using siRNA mediated depletion, immunofluorescence and western blotting (**Supplementary Figure S1B - D**). We apologize for not making this point clearer in our manuscript. However, in our submitted manuscript we had addressed this concern because we included stainings with two additional APC/C antibodies – APC3 and APC8 (**Supplementary Figure S1A**). We have revised the text to more clearly direct the reader to these experiments.
We also refined our text to emphasize that it is very well established that core APC/C subunits are rarely present on their own and that therefore the detection of three individual APC/C core subunits at the centrosome by immunofluorescence establishes the presence of the whole complex (Vodermaier, 2003, Current Biology; Passmore, 2004, Biochem Soc Trans.; Schwickart, 2004, Mol Cell Biol; Ohi, 2007, MolCell). These data are also supported by our western blot analysis, where we often used two different APC/C antibodies in the same experiment.

© 2021. Published by The Company of Biologists under the terms of the Creative Commons Attribution License (https://creativecommons.org/licenses/by/4.0/).

3) Notably missing from this work is a co-localisation between APC/C and Cep152. Considering these are the main molecules studied here (and their physical and functional interplay), this is a must. One would expect at least a partial co-localisation between them, considering that they are suggested to interact and one to be a substrate of the other.

This experiment is a very important suggestion. However, since both reliable and effective antibodies against APC2 and Cep152 are produced in rabbit, which makes direct co-staining difficult, we used our inducible eGFP-Cep152 cell line, and stained APC2 in these cells. As expected, (eGFP-)Cep152 and APC2 show a high degree of co-localization. We included these new data in **Figure 3E**.

4) The most downstream and most-important PCM component (at least in terms of MT-nucleation) is gamma-Tubulin (complexes). So I think the authors need to use anti-gamma-Tubulin Ab in all the experiments where they study/quantify the effects on PCM-recruitment. Not instead of PCNT, but in addition to.

We performed the experiment as suggested. We included gamma-tubulin as well as Cdk5rap2/Cep215 in our analysis. Cdk5rap2 is recruited to the centrosome by PCNT (Kim and Rhee, 2014, PLOS One; Pagan et al, 2014, Nature Cell Biology) and has itself been implicated in recruiting gamma-tubulin to the centrosome. In the presence of stabilized eGFP-Cep152^{DBK}, Cdk5rap2 intensity at the centrosome is reduced (new **Supplementary Figure 6A**), however to a lesser degree compared with PCNT. The same trend is observed for gamma-tubulin (new **Supplementary Figure 6A**). We thank the reviewer for this excellent suggestion, as these new observations are consistent with our data on PCNT.

5) In several places where immunoblots are used for drawing conclusions on comparative strength of interactions or levels of ubiquitination, there is no quantification provided. This should be corrected and normalisation by inputs should be done where relevant. The figures in question are Fig. 3C; Fig. 5C; Suppl.Fig. 5D. Please, check for others I may have missed.

We thank the reviewer for pointing out this oversight. We corrected this and now provide quantifications for all immunoblots where differences are mentioned in the text. As the reviewer identified, these are **Figures 3C, 5C and Supplementary Figure 5D**.

Minor points:

1) Page 5: In several places the descriptive “APC/C molecules” is used. If I understand correctly, the authors mean instead the whole multimolecular APC/C scaffold/complex, which cannot be referred to as a “molecule”. Please, correct it.

We agree that this wording might be confusing for readers. We removed it accordingly.

2) Page 7: “To generate a small-scale interaction network…”. Use instead “dependency/hierarchy network” as this is what you are testing for, not interactions.

We thank the reviewer for this suggestion. We decided to use dependency network instead.

3) Page 8: “A concentration of 200 ng/mL tetracycline…”. In all the figures pg/ml is used. Which one is correct?

We corrected this mistake. The concentration that was used throughout the study was 200 pg/mL tetracycline.

4) Page 10: “…less microtubules…” Correct to “…fewer microtubules…”
We thank the reviewer again for this. Grammar now corrected.

© 2021. Published by The Company of Biologists under the terms of the Creative Commons Attribution License (https://creativecommons.org/licenses/by/4.0/).

Second decision letter

MS ID#: JOCES/2021/259273

MS TITLE: The APC/C targets the Cep152-Cep63 complex at the centrosome to regulate mitotic spindle assembly

AUTHORS: Thomas Tischer, Jing Yang, and David Barford
ARTICLE TYPE: Research Article

I am happy to tell you that your manuscript has been accepted for publication in Journal of Cell Science, pending standard ethics checks.

Reviewer 1

Advance summary and potential significance to field

Tischer et al. studied the role of the cell cycle ubiquitin ligase APC/C in the regulation of centrosomes and spindle assembly in cultured human cells. It was shown that some of APC/C subunits are localised near the centrosome, accumulating during mitosis. This localisation depends on a conserved centrosome component CEP152. CEP152 and APC/C interacted with each other; whether the interaction is direct or indirect is not determined. The authors also provide evidence that CEP152 is also a substrate of APC/C-dependent ubiquitination. The mutant forms of CEP152, which had its putative APC/C degrons mutated, accumulates at mitotic centrosomes and causes a delay in mitotic progression, accompanying chromosome misalignment and lagging chromosomes. Furthermore, in this condition, the interaction between CEP57 and PCNT is reduced, leading to a model where the ubiquitination of CEP152 by APC/C allows CEP57 to dissociate from the CEP152-CEP63 inhibitory complex and bind PCNT to form the spindle. Based on these observations the authors propose that APC/C regulates spindle assembly by promoting the microtubule nucleation activity of the centrosome through the regulation of CEP152 levels at the centrosomes.
This study contains novel and important observations about centrosomes and cell cycle control mechanisms. In particular, the study provides new molecular insight into the mechanism that controls the conversion of the centrosome from the interphase form to the mitotic form as well as the spatial regulation of the functions of APC/C. Their proposed model for the role of CEP152 degradation in the conversion of the centrosome is straight-forward and quite attractive, which is well supported by their experimental data. I therefore support this paper for publication in JSC

Comments for the author

The authors have responded all of my comments properly. I am now in full support for the publication of this manuscript in JCS.

Reviewer 2

Advance summary and potential significance to field

The authors demonstrated that APC/C localizes to the centrosome during mitosis. The most notable discovery however is that they show evidence that Cep152 is responsible for recruiting APC/C to the centrosome in mitosis and is itself a substrate of APC/C.

I maintain my previous opinion that I support the publication of this paper in JCS. I supported this also in the former version, but as it is also improved by addressing other reviewers questions, I even more support it.

© 2021. Published by The Company of Biologists under the terms of the Creative Commons Attribution License (https://creativecommons.org/licenses/by/4.0/).

Comments for the author

The authors demonstrated that APC/C localizes to the centrosome during mitosis. The most notable discovery however is that they show evidence that Cep152 is responsible for recruiting APC/C to the centrosome in mitosis and is itself a substrate of APC/C.
I maintain my previous opinion that I support the publication of this paper in JCS. I supported this also in the former version, but as it is also improved by addressing other reviewers questions, I even more support it.

Reviewer 3

Advance summary and potential significance to field

The authors have addressed all my comments and suggestions appropriately in their revised manuscript. I consider it ready for publication in JCS.

Comments for the author

The authors have addressed all my comments and suggestions appropriately in their revised manuscript. I consider it ready for publication in JCS.

© 2021. Published by The Company of Biologists under the terms of the Creative Commons Attribution License (https://creativecommons.org/licenses/by/4.0/).
